# Low-Temperature Decomposition of CaCO_3_ in NiO/CaCO_3_ and CuO/CaCO_3_ Powder Under H_2_ at Industrial Waste Heat Temperatures

**DOI:** 10.3390/ma19173795

**Published:** 2026-09-06

**Authors:** Tomoaki Yoshida, Ryota Gemma

**Affiliations:** 1 Graduate School of Science and Technology, Tokai University, Hiratsuka 259-1292, Japan; 2 Micro/Nano Technology Center, Tokai University, Hiratsuka 259-1292, Japan

**Keywords:** CO_2_ utilization, Ni catalyst, CaCO_3_, CO_2_ methanation, calcium looping

## Abstract

Calcium looping (CaL) is a CO_2_ capture technology based on the reversible carbonation of calcium salts. Calcium-based materials are highly reactive with CO_2_ and are generated as by-products or waste across a wide range of industries—such as cement, steel, and fisheries—leading to expectations that the calcium looping (CaL) process can be implemented at a low cost. However, a key challenge remains in the regeneration step: releasing CO_2_ from CaCO_3_, the carbonated form of the sorbent, and returning it to a reusable state conventionally requires calcination at around 700 °C, which imposes a substantial energy penalty. This study investigates the decomposition of solid-phase calcium carbonate (CaCO_3_) using Ni-supported composites under a hydrogen atmosphere at industrial waste heat temperatures. TPD-MS confirmed CO_2_ release below 250 °C, far lower than 700 °C, the typical decomposition temperature of CaCO_3_. The XRD and XPS results showed that CaCO_3_ was partially converted to Ca(OH)_2_ in the surface region. In situ DRIFTS measurements revealed a decrease in the CO_3_^2−^ signal upon heating metal oxide/CaCO_3_ composites under a hydrogen atmosphere. The magnitude of decrease in the CO_3_^2−^ signal was significant in Ni-loaded CaCO_3_ compared to Cu-loaded CaCO_3_, suggesting a difference in hydrogen dissociation ability of loaded metals.

## 1. Introduction

Global warming continues to intensify, with CO_2_ recognized as a major contributing factor, primarily owing to emissions from combustion of fossil fuels [1]. Accordingly, countries worldwide are pursuing efforts to reduce CO_2_ emissions through the exploration of alternative energy sources and the development of CO_2_ capture technologies.

Carbon Capture, Utilization, and Storage (CCUS) is a collective term for technologies that separate, capture, store, and utilize CO_2_ from gases emitted by fossil fuel combustion facilities, such as thermal power plants and factories; it is attracting attention as an effective measure against global warming [2,3].

The calcium looping (CaL) process is one of such CCUS technologies [2,4,5], in which CO_2_ is reacted with CaO and chemically fixed as CaCO_3_, as shown in Equation (1), followed by thermal decomposition to separate and recover CO_2_.

(1) $$CaO+{CO}_{2}\to {CaCO}_{3}\;\;\;\;{\;∆H}_{298\;K}^{o}=-178\;kJ/mol$$

CaO exhibits high reactivity toward CO_2_ and reacts even at room temperature. Furthermore, the addition of water generates Ca(OH)_2_, which further enhances reactivity [6] (Equations (2) and (3)).

(2) $$CaO+\;H_{2}O\to {Ca(OH)}_{2}\;\;\;\;\;\Delta H_{298\;K}^{o}=-67.3\;kJ/mol$$

(3) $${{Ca(OH)}_{2}\;+{CO}_{2}\to CaCO}_{3}\;\;\;\;\;\Delta H_{298\;K}^{o}=-113\;kJ/mol$$

Calcium salts are abundantly available as industrial wastes, including shells from shellfish aquaculture, slag from the steel industry, and residues from concrete plants, making them highly attractive materials due to their extremely low cost and recycling potential [7].

However, the thermal decomposition reaction of CaCO_3_ (the reversal of Equation (1)) requires high temperatures above 700 °C. This poses a significant drawback when attempting to supply the necessary heat from industrial sectors, as the range of applicable industries offering such high temperatures is limited. Accordingly, lowering the CaCO_3_ decomposition temperature is desired in the first place.

Currently, the majority of unutilized waste heat released in industry falls within the temperature range below 300 °C [8]. If the decomposition temperature could be reduced to this range, the number of industries capable of utilizing this CaL process would be expected to increase substantially. The decomposition of CaCO_3_ thermodynamically requires the CO_2_ partial pressure to fall below the equilibrium pressure. Therefore, reducing the CO_2_ partial pressure enables decomposition at lower temperatures.

Previously, Valverde et al. demonstrated using in situ XRD that the thermal decomposition of CaCO_3_ under a helium atmosphere is completed approximately at 725 °C and also investigated its cyclability [9]. Other relevant studies reported the decomposition of natural limestone under reduced pressure [10], and the results of kinetic investigations [11], and an activation energy for the decomposition reaction of 92.58 kJ/mol in the temperature range of 700–850 °C.

The catalytic effect of water vapor on CaCO_3_ decomposition has been recognized since Berger [12], MacIntire and Stansel [13]; however, clarification of the underlying mechanism, including the formation of HCO_3_^−^, was not achieved until the report by Giammaria et al. in 2019 [14] and the study by Donglin He et al. in 2020 [15].

It has also been reported that heating under a hydrogen atmosphere lowers the CO_2_ partial pressure, reducing the decomposition temperature by approximately 120 °C (from ~900 °C to ~786 °C) [16]. Moreover, it has been reported that loading Ni and heating under a hydrogen atmosphere enables decomposition at approximately 500 °C with direct CH_4_ generation rather than CO_2_ release [17,18].

Nevertheless, in all cases, reports of thermal decomposition below the target temperature of 300 °C remain limited to examples employing NaOH and similar reagents [19,20] or electrochemical approaches [21], and significant challenges remain for the widespread adoption of CaL.

In the present study, inspired by (1) the catalytic effect of water vapor [12,13,14,15], (2) direct methanation of CaCO_3_ under hydrogen atmosphere at approximately 500 °C [17,18], and (3) reduction in CaCO_3_ decomposition temperature via CO_2_ partial pressure decrease under hydrogen atmosphere [9], we attempted to lower the decomposition temperature of CaCO_3_ by exploiting these effects in combination.

Specifically, CaCO_3_ powder loaded with NiO or CuO was prepared and heated under a H_2_ atmosphere, and the following three impacts are expected: (1) H_2_O supply through NiO reduction, (2) supply of atomic H from Ni [22], and (3) reduction in CO_2_ partial pressure under H_2_ atmosphere. Several in situ approaches were employed to investigate the possibility of thermal decomposition of CaCO_3_ at temperatures below 300 °C.

## 2. Experimental Methods

### 2.1. Sample Preparation

Nickel acetate tetrahydrate (Fujifilm Wako, Osaka, Japan, Ni(CH_3_COO)_2_·4H_2_O, purity 99.9%) and calcium carbonate (Kojundo Kagaku, Saitama, Japan, CaCO_3_, purity 99.9%) were weighed to achieve the prescribed Ni:Ca molar ratios (see Table 1), dissolved in distilled water, and evaporated to dryness under stirring at 110 °C. After drying, the mixture was calcined in air at 500 °C for 2 h to obtain NiO/CaCO_3_ composite powder. Since Cu, unlike Ni, exhibits a high activation barrier toward H_2_ dissociation on all but specific crystal facets [23], copper acetate (Fujifilm Wako, Cu(CH_3_COO)_2_·H_2_O, purity 99.9%) and calcium carbonate were processed using the same procedure to prepare CuO/CaCO_3_ composite powder to compare the results with NiO/CaCO_3_.

Samples for DRIFTS were prepared with a M:Ca molar ratio of 1:1 using calcium acetate monohydrate, (Fujifilm Wako, Ca(CH_3_COO)_2_ H_2_O, purity 99.0%), as the Ca source in order to further enhance the dispersion of the metal oxide/CaCO_3_.

### 2.2. Temperature-Programmed Desorption–Mass Spectrometry (TPD-MS)

**Figure 1:**
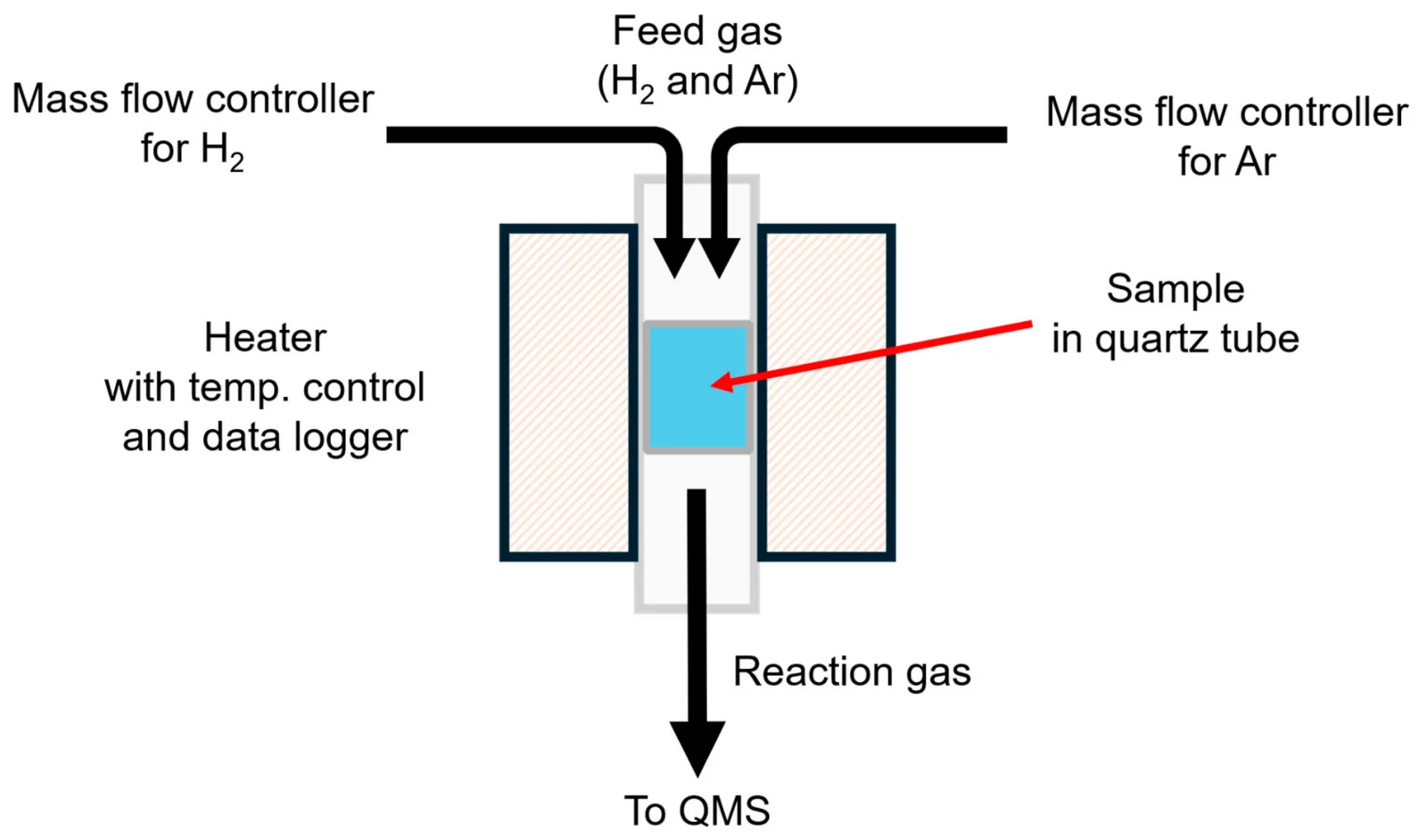
Schematic diagram of a home-built fixed-bed reactor (FBR) with a Quadrupole Mass Spectrometer for the TPD-MS (mass flow controller for H_2_: KOFLOC D8500MC-0-I, mass flow controller for Ar: KOFLOC 3660 series (control unit: CR-300), QMS: PFEIFFER QMG250 Prisma Pro).

A home-built fixed-bed catalytic reactor (FBR, Figure 1) was loaded with 0.2 g of sample powder, and the temperature was increased to 250 °C at a rate of 10 °C/min under Ar gas flow (10 ccm) controlled by a mass flow controller (MFC: KOFLOC, Kyoto, Japan, 3660 series (control unit: CR-300). Upon reaching 250 °C, the Ar gas flow was stopped, H_2_ (20–40 ccm) was introduced via a mass flow controller (MFC: KOFLOC D8500MC-0-I), and the heating was continued while maintaining the temperature. The composition of the reactor outlet gas was monitored in real time by a Quadrupole Mass Spectrometer (QMS: PFEIFFER, Asslar, Germany, QMG250 Prisma Pro). The powder samples before and after the heating treatment were subjected to the various characterization methods described below.

### 2.3. Sample Characterization

#### 2.3.1. Electron Probe Microanalyzer (EPMA) and Scanning Electron Microscopy–Energy-Dispersive X-Ray Spectroscopy (SEM-EDS)

Confirmation of MO/CaCO_3_ composite formation in the prepared samples was performed using an electron probe microanalyzer (EPMA: Shimadzu, Kyoto, Japan EPMA-8050G) and a field-emission scanning electron microscope equipped with energy-dispersive X-ray spectroscopy (FE-SEM/EDX: JEOL, Tokyo, Japan, JSM-7100F/JED-2300F).

#### 2.3.2. X-Ray Fluorescence (XRF)

Elemental composition (elemental ratio) of the samples was semi-quantitatively determined using an X-ray fluorescence spectrometer (XRF: Hitachi, Tokyo, Japan, EA1000A III). The elemental ratios were calculated from the obtained fluorescence intensities based on a semi-quantitative (standardless) analysis method.

#### 2.3.3. Powder X-Ray Diffraction (XRD)

Phase identification of the samples before and after the heating treatment was performed using an X-ray diffractometer (XRD: Rigaku MiniFlex600, Cu Kα radiation, λ = 0.15418 nm). Measurements were conducted over the range of 2θ = 20–60°, and phase identification was carried out from the obtained diffraction patterns. The observed diffraction patterns were identified by comparison with patterns simulated from the crystal structure data of CaCO_3_ [24], Ca(OH)_2_ [25], CaO [26], NiO [27], Ni [28], CuO [29] and Cu [30].

#### 2.3.4. X-Ray Photoelectron Spectroscopy (XPS)

Changes in the surface chemical bonding state of each sample were evaluated using an X-ray photoelectron spectrometer (XPS: ULVAC-PHI, Kanagawa, Japan, PHI Quantera II, Al Kα radiation, hν = 1486.6 eV). Peak deconvolution was performed on the Ca 2p3/2 spectral region to estimate the contributions attributed to CaCO_3_ and Ca(OH)_2_. Binding energies were referenced to C 1s (C=C: 284.8 eV) for peak identification.

XPS probes only the near-surface region, and this fraction does not represent the bulk conversion.

#### 2.3.5. In Situ DRIFTS Measurements

To track the temporal evolution of the CO_3_^2−^ absorption peak derived from CaCO_3_ in IR spectra, in situ measurements were performed using diffuse reflectance infrared Fourier transform spectroscopy (DRIFTS: Shimadzu IRaffinity-1S/PIKE Technologies, Medison, WI, USA, diffuse reflectance accessory). After heating the sample to 250 °C under Ar gas flow, the intensity of the characteristic absorption band of CO_3_^2−^ (approximately 1791 cm^−1^) was measured as a function of time under H_2_ gas flow at 40 ccm.

#### 2.3.6. Thermogravimetric Analysis (TG)

The thermal decomposition behavior of NiO/CaCO_3_ composite powder under a hydrogen atmosphere was evaluated using a thermogravimetric analyzer (TG: Seiko Instruments Inc., Chiba, Japan, TG/DTA 6200). The sample was heated from room temperature to 850 °C at a rate of 10 °C/min under a flow of Ar–H_2_ mixed gas (H_2_ 10 vol%) at 200 ccm, and the weight change was recorded continuously. For comparison, measurements under the same conditions in an Ar atmosphere were also performed.

## 3. Results and Discussion

### 3.1. Characterization of As-Prepared Samples

The EPMA, XRF, and TG results were all consistent with the intended composition, confirming that the samples were prepared as designed. The differences described in the following sections can therefore be attributed to the type of supported metal oxide rather than to variations in sample preparation.

**Figure 2:**
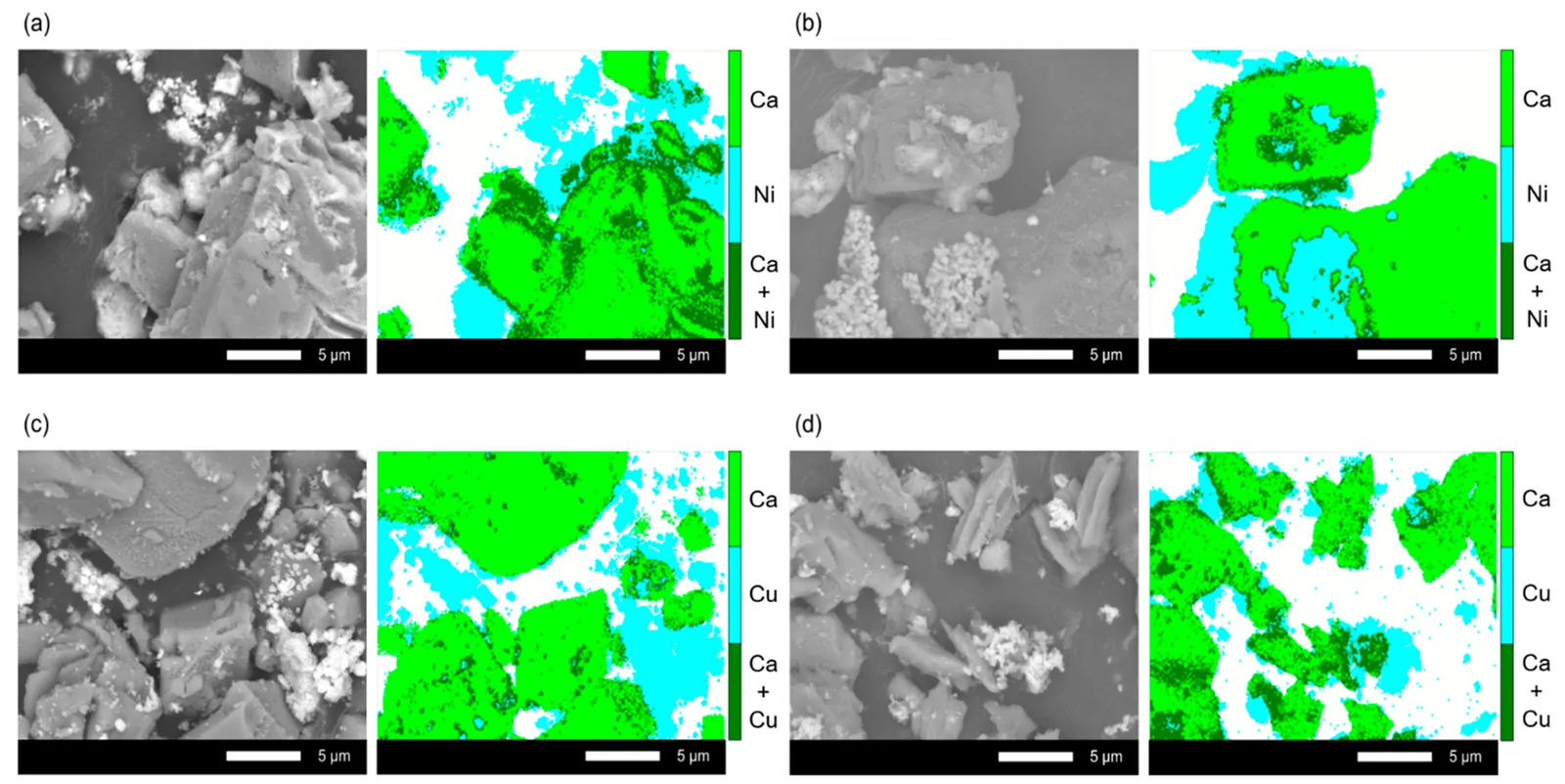
Secondary electron (SE) image and elemental maps of the samples by EPMA (Ca: light green, supported metal element: light blue, overlap of Ca and metal-supported element: dark green); (**a**) NiO/CaCO_3_ (1:9), (**b**) NiO/CaCO_3_ (2:8), (**c**) CuO/CaCO_3_ (1:9), (**d**) CuO/CaCO_3_ (2:8).

Figure 2 shows elemental maps obtained by EPMA for the prepared samples. In both the NiO/CaCO_3_ and CuO/CaCO_3_ samples, although heterogeneous distributions were observed, the presence of the loaded elements in the vicinity of the CaCO_3_ particles was confirmed. However, because the sample surfaces were not flat, accurate quantification of elemental ratios by EPMA and SEM-EDS was difficult. Therefore, the presence of CaCO_3_ at the prescribed compositional ratio was verified by the weight loss associated with CaCO_3_ thermal decomposition measured by TG under Ar flow (Figure S1). Table 2 summarizes the contents of the supported elements and Ca determined by XRF for each sample, together with the nominal values calculated from the amounts charged during preparation. The measured Ni and Cu contents are higher than the corresponding nominal values. This discrepancy is partly due to the error inherent in semi-quantitative XRF analysis. Another possible cause is the structure of the composites. As shown by the EPMA results in Figure 2, the supported elements are enriched at the sample surface rather than being uniformly distributed throughout the composite. Since XRF collects information only from a limited depth below the surface, such surface-enriched elements are overestimated. Furthermore, TG measurements under Ar flow showed no appreciable differences in the weight change ratios among the samples (Figure S1 and Table S1). We therefore conclude that the samples have the intended loading amounts. Unless otherwise stated, the results presented in the following sections pertain to samples with a metal oxide-to-CaCO_3_ weight ratio of 1:9.

### 3.2. Solid-Phase Changes During H_2_ Treatment (XRD, XPS and in Situ DRIFTS)

**Figure 3:**
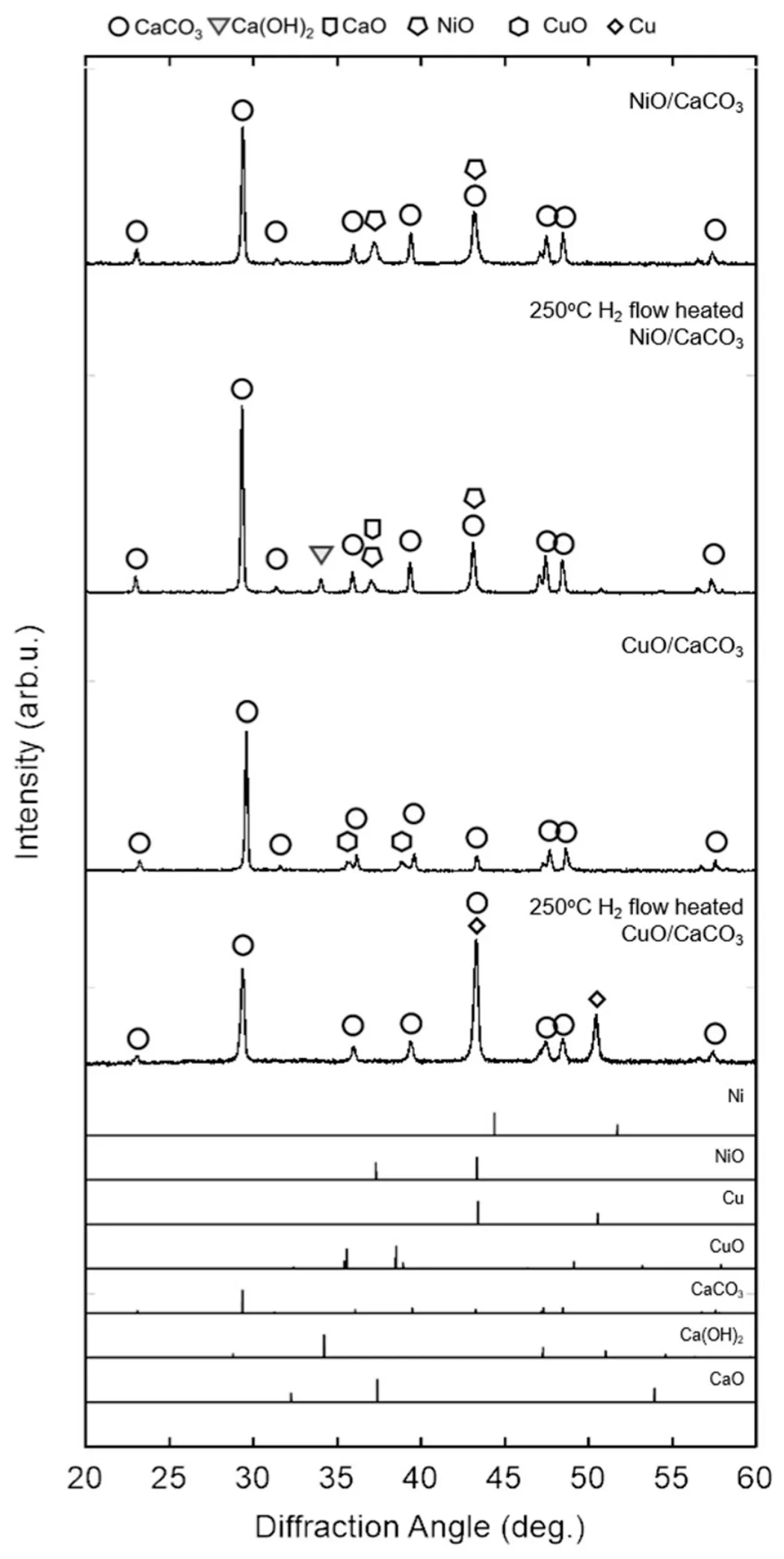
XRD patterns of NiO/CaCO_3_ (1:9) and CuO/CaCO_3_ (1:9) composite powders before and after heating at 250 °C for 2 h under H_2_ atmosphere (Cu Kα: λ = 0.15418 nm). Simulated patterns of CaCO_3_ [24], Ca(OH)_2_ [25], CaO [26], NiO [27], Ni [28], CuO [29] and Cu [30] are shown at the bottom.

Figure 3 shows the XRD results for NiO/CaCO_3_ (1:9) and CuO/CaCO_3_ (1:9) after heating at 250 °C under a hydrogen atmosphere. In the results for the NiO/CaCO_3_ powder, a peak attributable to Ca(OH)_2_ (101) was confirmed near 2θ = 34°, presumably arising from CaO generated by CaCO_3_ decomposition reacting with co-produced water vapor upon the oxide reduction. Regarding the Ni species, although the peak intensities of NiO (200) and NiO (111) decreased, several peaks of NiO remained, indicating that the reduction was not complete. Consequently, evaluation of the presence of CaO was difficult due to peak overlap between NiO (111) and CaO (200). In contrast, the only change observed in CuO/CaCO_3_ was the appearance of Cu peaks associated with CuO reduction. No clear changes were observed in the calcium species, such as the appearance of Ca(OH)_2_ (101) or CaO (200) peaks, with CaCO_3_ as remained. However, a decrease in the peak intensity of CaCO_3_ (104) was confirmed, suggesting a reduction in crystallinity or partial decomposition of CaCO_3_.

**Figure 4:**
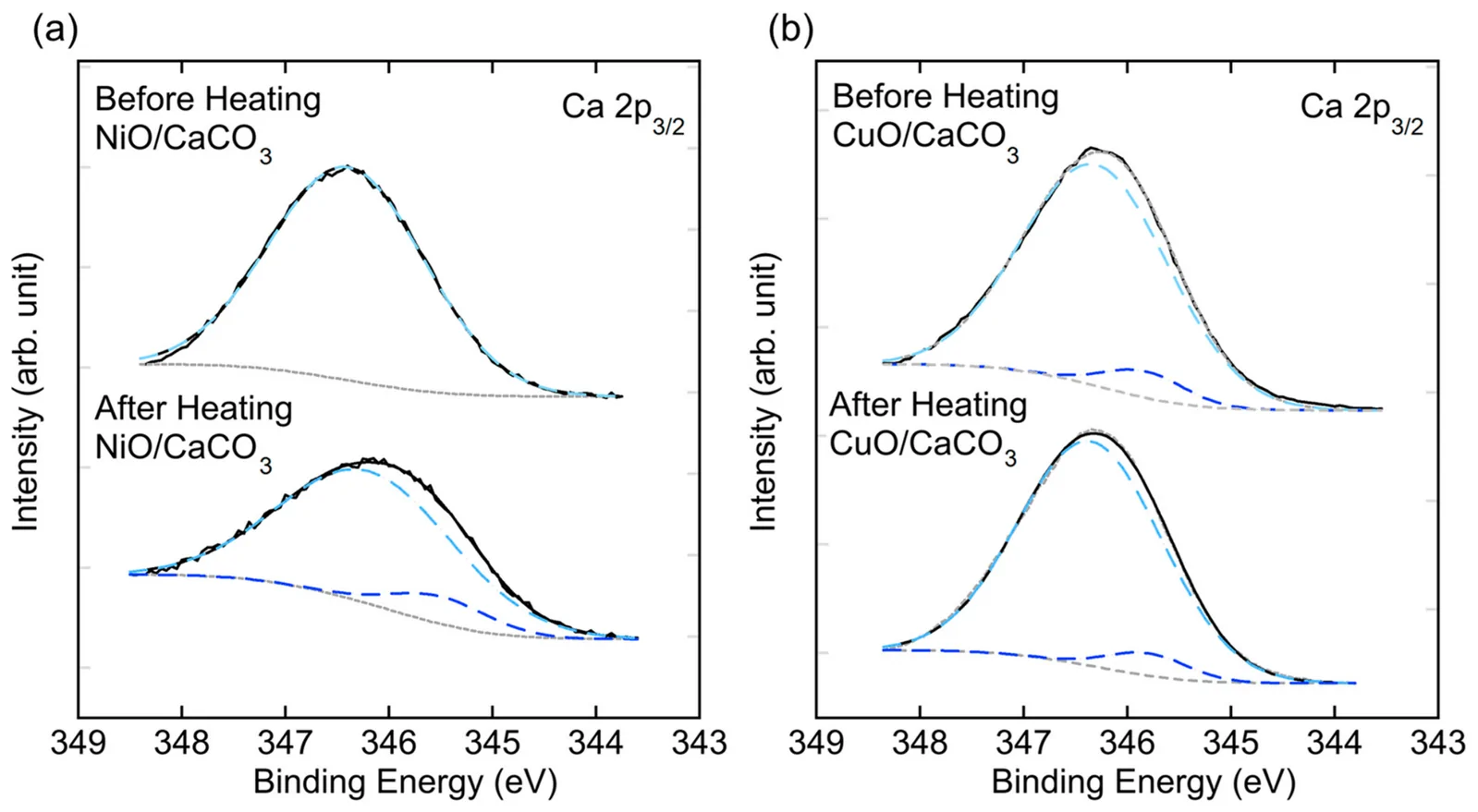
Ca 2p_3/2_ XPS spectra of (**a**) NiO/CaCO_3_ and (**b**) CuO/CaCO_3_ composite powders before and after heating at 250 °C for 2 h under H_2_ atmosphere (metal oxide-to-CaCO_3_ weight ratio of 1:9). Solid lines represent the measured data, and dashed lines represent the fitted curves (blue lines) and the background (gray line).

To further evaluate these findings, XPS analysis of the Ca 2p_3/2_ region was performed for each sample (Figure 4). Before heating under a hydrogen atmosphere, NiO/CaCO_3_ could be described by a single fitting curve corresponding to CaCO_3_. After heating under a hydrogen atmosphere, however, the spectrum could not be explained by fewer than two peaks, suggesting that approximately 11% of the sample surface had been converted to Ca(OH)_2_. Note that this fraction reflects only the near-surface region probed by XPS and cannot be translated into a bulk conversion. This second component appeared at a binding energy lower than that of CaCO_3_. Since the Ca 2p spectrum alone does not allow Ca(OH)_2_ and CaO to be distinguished, the assignment to Ca(OH)_2_ is based on the concurrent appearance of the Ca(OH)_2_ (101) reflection in XRD (Figure 3). The O 1s spectrum was not used for this assignment since contributions from the supported Ni species overlap with those of the Ca species. In contrast, for CuO/CaCO_3_, although neither the pre- nor post-experiment spectra could be described by a single peak, the difference revealed by peak fitting was extremely small. It is therefore considered that either decomposition of CaCO_3_ occurred to only a negligible extent, or that recarbonation occurred after the experiment, converting the product back to CaCO_3_ at the time of analysis.

The chemical states of the Ni and Cu species were also examined by XPS (Figure S3). Given the complexity of the Ni 2p spectra of NiO [31], the Ni species were evaluated from the separation between the main peak and its shake-up satellite, which suggested the coexistence of NiO and metallic Ni after the treatment in the NiO/CaCO_3_ system. For the CuO/CaCO_3_ system, the almost complete disappearance of the Cu^2+^ shake-up satellite [32] suggested the reduction in CuO.

**Figure 5:**
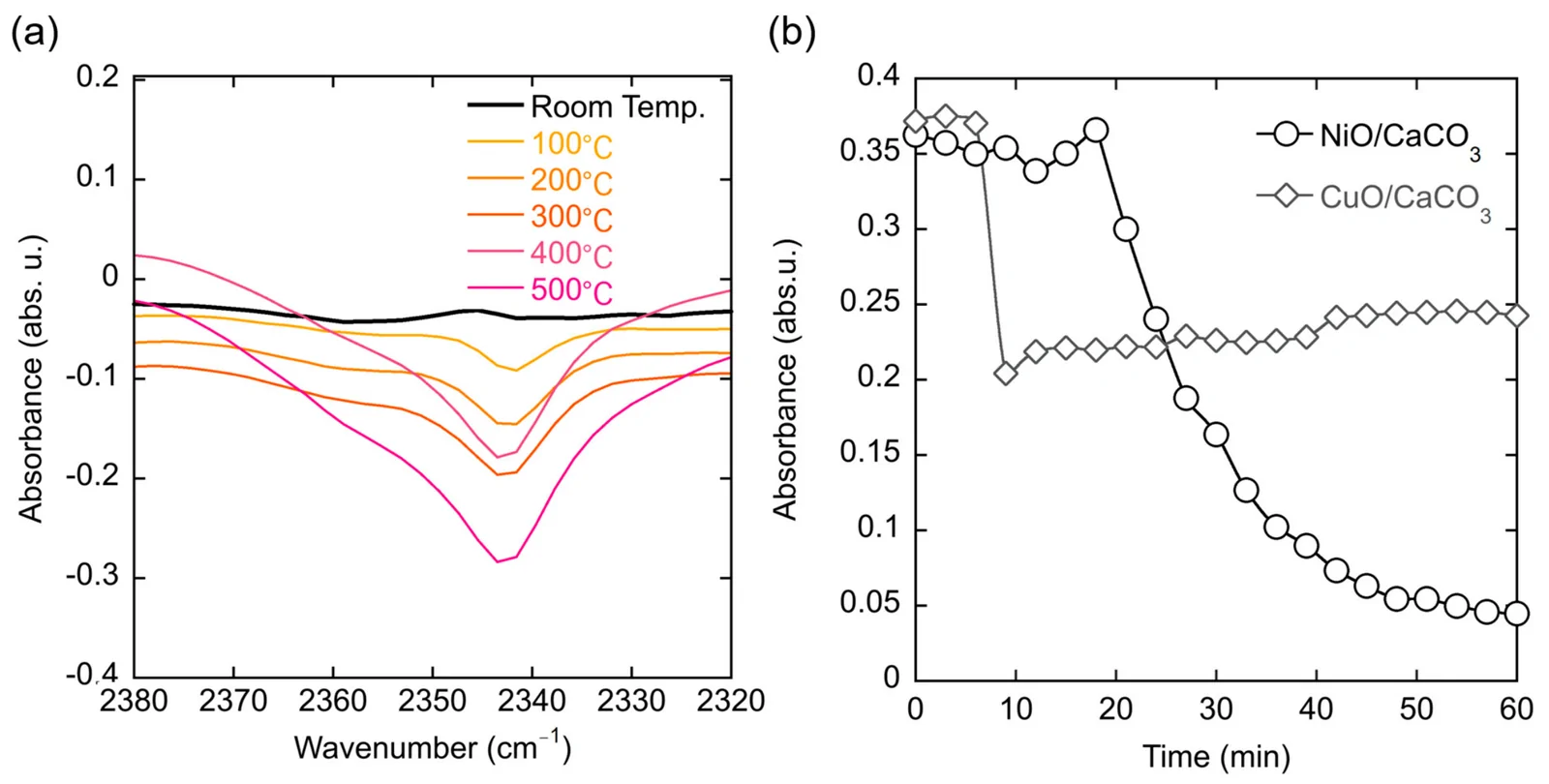
In situ DRIFTS measurements of NiO/CaCO_3_ and CuO/CaCO_3_ during heating under flowing H_2_ (40 ccm). (**a**) Temperature dependence of the gas-phase CO_2_ absorbance at around 2344 cm^−1^ (NiO/CaCO_3_ sample, Ni:Ca elemental ratio of 1:1). (**b**) Time course of the CO_3_^2−^ absorbance at 1791 cm^−1^ at a constant sample temperature of 250 °C under H_2_; t = 0 corresponds to the onset of H_2_ introduction.

Figure 5a shows temperature-dependent absorbance change in gas-phase CO_2_ in DRIFTS during heating to 500 °C under H_2_ gas at 40 ccm for a NiO/CaCO_3_ sample with a Ni:Ca elemental ratio of 1:1. The powder was diluted 10-fold by weight using calcium fluoride. A gradual increase in CO_2_ was observed from 100 °C onward. This might suggest that CO_2_ adsorbed on the surface began to be released upon heating at approximately 100 °C. Once released, CO_2_, however, would be removed from the system by continuous H_2_ gas flow during the DRIFTS. This is opposite to the observation demonstrating significant absorbance by gas-phase CO_2_ over a wide temperature range. Therefore, the sustained signal of CO_2_ in the DRIFTS spectra suggests continuous CO_2_ generation upon heating to 500 °C.

Figure 5b shows the temporal change in the CO_3_^2−^ peak at 1791 cm^−1^ after switching from Ar to H_2_ flow (40 ccm) for undiluted samples of the same composition that had been heated to 250 °C under Ar flow. A baseline was drawn using reference points at 1780 cm^−1^ and 1800 cm^−1^. In both samples, the absorbance decreased with time. In the case of the NiO/CaCO_3_ system the absorbance of CO_3_^2−^ gradually decreased from 0.35 to 0.05 in 60 min and nearly saturated. In contrast, the CuO/CaCO_3_ system dropped only to 0.2 within 10 min and kept nearly the same absorbance.

This difference might be due to the different IR reflectivity of Ni and Cu. Using the closest reported value of 1774 cm^−1^ (λ = 5.62 µm) to 1791 cm^−1^ from the reference data handbook [33], the reflectivities of Ni and Cu were calculated using the Fresnel equation (Equation (4)), yielding values of 98% for Cu and 96% for Ni, respectively. Even considering that these are for normal incidence and should be regarded strictly as approximate values, the difference in IR reflectivity is negligible.

(4) $$R=\frac{{\left(n-1\right)}^{2}+k^{2}}{{\left(n+1\right)}^{2}+k^{2}}$$

(R: reflectivity, n: refractive index, k: extinction coefficient)

The result therefore suggests that the observed difference between these two samples in Figure 5b is not from optical artifacts. Furthermore, in contrast to Ni, which has been shown to readily dissociate and adsorb H_2_ [22,34], both DFT calculations and experimental studies have demonstrated that Cu exhibits a high activation barrier for H_2_ dissociation over the majority of its surface [23]. Based on these findings, the difference in the change in the 1791 cm^−1^ band observed in the low-temperature decomposition of CaCO_3_ under hydrogen atmosphere for NiO and CuO systems could primarily be due to the influence of atomic H supplied from reduced Ni, in addition to the reaction being driven by H_2_O generated upon reduction in NiO and CuO, as shown in Equations (5) and (6) (calculated from standard enthalpies of formation; the NBS Tables of Chemical Thermodynamic Properties [35]), respectively.

(5) $$NiO+\;H_{2}\to Ni+\;H_{2}O\;\;\;\;\;\Delta H_{298\;K}^{o}=-2.1\;kJ/mol$$

(6) $$CuO+\;H_{2}\to Cu+\;H_{2}O\;\;\;\;\;\Delta H_{298\;K}^{o}=-84.5\;kJ/mol$$

Even in the case of NiO/CaCO_3_, the peak of CO_3_^2−^ at 1791 cm^−1^ did not disappear completely, and the XRD and XPS results described above also indicate that a large proportion of CaCO_3_ remained after the experiment (Figure 3 and Figure 4). Given that no changes were observed when heating CaCO_3_ alone in a H_2_ atmosphere (Figures S2 and S8), this phenomenon is considered to be localized in the vicinity of the supported metal oxide and, in particular, dependent on the number density of NiO/CaCO_3_ interfaces.

**Figure 6:**
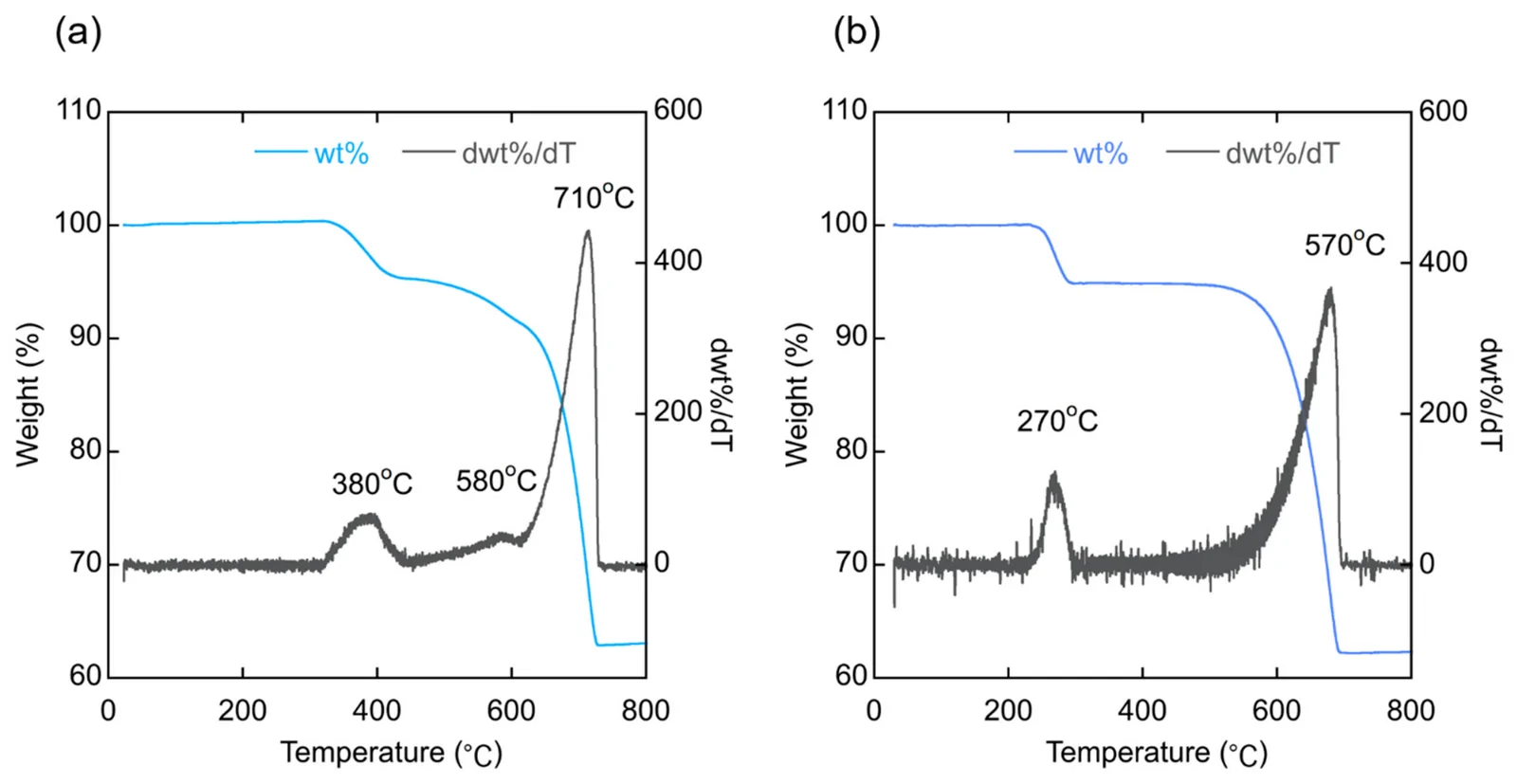
TG results of (**a**) NiO/CaCO_3_ (**b**) CuO/CaCO_3_ under Ar + H_2_ (H_2_ 10 vol.%), with metal oxide-to-CaCO_3_ weight ratio of 1:9. Weight change at 380 °C due to NiO reduction shows a slight shoulder peak in dwt%/dT, hinting at low-temperature CO_2_ release from NiO/CaCO_3_ under the presence of H_2_.

Figure 6a shows the weight change curve of the NiO/CaCO_3_ composite powder under Ar–H_2_ flow measured by TG. Weight change peaks were observed at 380 °C, 580 °C, and 710 °C. The event at 380 °C is inferred to involve a two-stage reaction, as the dwt%/dT curve shows the onset of weight change from approximately 330 °C accompanied by a shoulder peak. As shown in Figure S1, the thermal decomposition behavior of this sample under Ar atmosphere consists of only a single weight change step corresponding solely to CaCO_3_ decomposition. Under H_2_, however, in addition to NiO reduction at 330 °C (Equation (5)) and CaCO_3_ thermal decomposition at 690 °C (Equation (1)), minor weight change events are also observed at the 330 °C shoulder and at 580 °C, which could be attributed to CO_2_ release and direct CH_4_ generation [17,18], respectively.

Figure 6b shows the results obtained under the same conditions using CuO/CaCO_3_ composite powder. Weight change events were confirmed at 280 °C and 580 °C, attributed respectively to the weight loss associated with the reduction in CuO by hydrogen and the weight loss associated with the thermal decomposition of CaCO_3_ at higher temperatures.

Padeste et al. [36] reported CaCO_3_ decomposition at approximately 300 °C by TG-MS study on CaCO_3_ loaded with transition metals under a hydrogen atmosphere. According to their report, in the case of Fe-loading, H_2_O and CO_2_ were released during the reduction in iron oxide occurring near 300 °C, while in the case of Ni, the fragment ion CH_3_^+^, which is characteristic of CH_4_, was detected.

To examine this further, TG measurements were performed by heating to 250 °C under Ar flow, followed by switching to Ar–10% H_2_ gas; however, weight changes beyond those attributable to NiO reduction were only marginal, and TG alone was not sufficient to verify this (Figure S3). Thus, QMS analysis was performed as described in the next section.

### 3.3. Gas Evolution Behavior Under H_2_ Monitored by QMS

The TG results suggested that weight loss occurred well below the decomposition temperature of CaCO_3_. The origin of this weight loss could mainly be due to the reduction in NiO or CuO. However, the XRD, XPS and in situ DRIFTS results suggest that the decomposition of CaCO_3_ might have occurred as well even at lower temperatures like 250 °C under H_2_.

**Figure 7:**
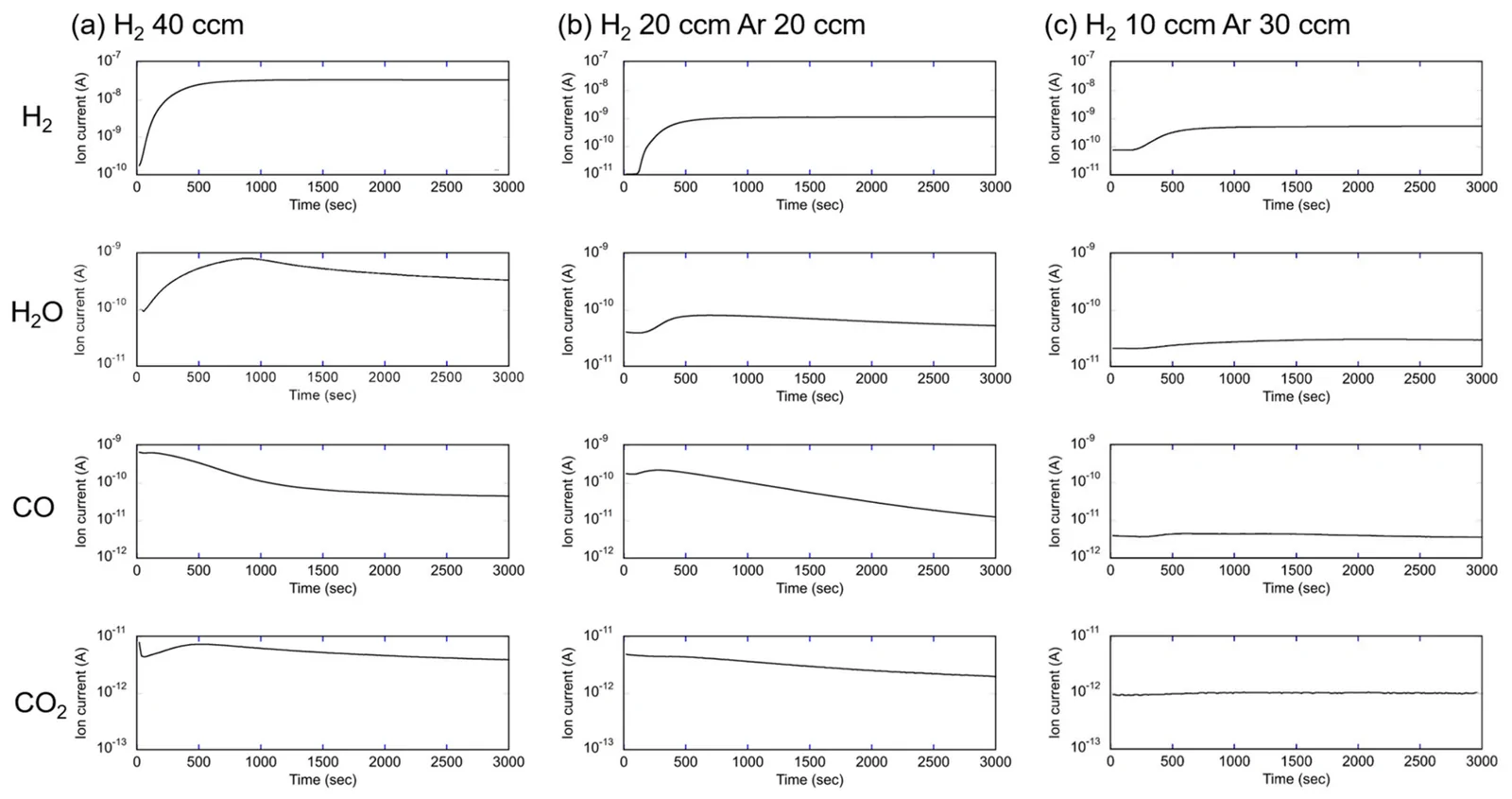
QMS analysis of reaction gas during temperature-programmed reduction (TPR) in NiO/CaCO_3_ at a constant temperature of 250 °C after switching to pure H_2_ or H_2_ + Ar mixed atmosphere from pure Ar flow. (**a**) H_2_ 40 ccm, Ar 0 ccm (1 atm H_2_) (**b**) H_2_ 20 ccm, Ar 20 ccm (0.5 atm H_2_) (**c**) H_2_ 10 ccm, Ar 30 ccm (0.25 atm H_2_). Note the remarkable increase in H_2_O signal, e.g., in (**a**) right after switching the flow gas from Ar to H_2_. CO_2_ signal also increases, following the increase in H_2_O signal.

To elucidate this further, QMS analysis under H_2_ at 250 °C was conducted using a fixed-bed catalytic reactor. NiO/CaCO_3_ (NiO:CaCO_3_ = 1:9 in weight) was loaded into the reactor and heated to 250 °C under Ar gas flow (40 ccm), after which the Ar flow was stopped and the gas was switched to H_2_ (40 ccm), with the outlet gas analyzed by QMS (Figure 7a).

Simultaneously with the onset of H_2_ flow, a rise in the H_2_O signal was observed, consistent with the expected NiO reduction reaction. A concomitant rise in the CO_2_ signal was also confirmed. Here, the simultaneous detection of H_2_O and CO_2_ suggests that H_2_O generated by NiO reduction promotes the decomposition of CaCO_3_. Subsequently, as the H_2_O signal decreased and the NiO reduction was completed, the CO_2_ signal also gradually decreased. This behavior is in line with the finding that the H_2_O supplied through NiO reduction plays an important role in driving CaCO_3_ decomposition.

Next, to investigate the effect of H_2_ partial pressure, experiments were performed with varying H_2_ concentration by diluting with Ar while maintaining a total gas flow rate of 40 ccm (Figure 7b,c). As in the case of pure H_2_, H_2_O generation attributable to NiO reduction was confirmed upon switching to H_2_ flow, accompanied by a rise in both CO and CO_2_ signals. Under 100% H_2_ atmosphere, CO_2_ was detected as the primary product, while the CO signal showed only baseline drift-level changes. In contrast, under conditions where H_2_ was diluted with Ar, the CO signal rise became pronounced, and the CO/CO_2_ ratio shifted progressively toward CO dominance as the H_2_ concentration decreased (Figure S5). Furthermore, with decreasing H_2_ concentration, the magnitude of the rise above baseline for both CO and CO_2_ signals decreased. These results indicate that the extent of CaCO_3_ decomposition depends on the H_2_ partial pressure.

As a control experiment, gas analysis was performed in the same manner under pure Ar gas flow at 40 ccm without H_2_ (Figure S5e). No changes other than baseline drift were observed. This result clearly demonstrates that all signal changes observed in Figure 7 arise from the presence of H_2_.

Taken together, these results demonstrate that CaCO_3_ decomposition at 250 °C is promoted by H_2_ and the product selectivity varies with the H_2_ partial pressure. Under high H_2_ concentration conditions (Figure 7a, 1 atm H_2_), CO generated by CaCO_3_ decomposition is rapidly converted to CO_2_ via the Water–Gas Shift (WGS) reaction [37] on the Ni surface with H_2_O co-generated by NiO reduction (Equation (7)). Under low H_2_ concentration conditions (Figure 7c, 0.25 atm H_2_), on the other hand, less H_2_O is available, suppressing the WGS reaction; eventually CO accumulates as the primary detected gas, and CO_2_ is negligible. This dependence of CO/CO_2_ selectivity on H_2_ partial pressure is also consistent with thermodynamic considerations in that the position of the WGS equilibrium is governed by the H_2_O/H_2_ ratio, and the enhanced H_2_O supply accompanying accelerated NiO reduction under high H_2_ concentrations shifts the equilibrium toward CO_2_.

(7) $$CO+H_{2}O\to {CO}_{2}+H_{2}\;\;\;\;\;\Delta H_{298\;K}^{o}=-41.1\;kJ/mol$$

In light of previous works by T. Hayashi et al. and B. Shao et al. on direct methanation of CaCO_3_ near 500 °C [17,18], it is considered that CH_4_ generation from CaCO_3_ does not proceed via CO_2_ released by CaCO_3_ thermal decomposition, but rather that CH_4_ is generated directly from CaCO_3_, driven by atomic H supplied from Ni (Equation (8)).

(8) $${CaCO}_{3}+4H_{2}\to CaO+\;{CH}_{4}+2H_{2}O\;\;\;\;\;\Delta H_{298\;K}^{o}=+14.1\;kJ/mol$$

The ΔG for this reaction at 523 K is calculated to be +23.2 kJ/mol, and the equilibrium constant is K = 4.8 × 10^−3^, well below unity. Therefore, the reaction is not spontaneous. Nevertheless, since the number of moles of gas decreases from four to three in this reaction, the equilibrium conversion reaches approximately 27.5% even when a closed system at a total pressure of 1 atm is assumed. In an open system, however, a consistently high partial pressure of the reactant is maintained under H_2_ flow because H_2_ is the only gaseous species on the left-hand side in Equation (8). The equilibrium is thus shifted toward the right-hand side, suggesting that the reaction can proceed.

Since the reduction in NiO is suggested to start already at 250 °C (Figure S4), yielding a metallic Ni surface, the low-temperature decomposition of CaCO_3_ near 250 °C is inferred to proceed as follows:

1. H_2_O is generated by the reduction in the loaded metal oxide.
2. Atomic H supplied from the catalytically active metal—formed upon reduction and capable of H_2_ dissociation—migrates to the adjacent CaCO_3_ surface (H spillover).
3. H_2_O and atomic H promote the generation of HCO_3_^−^ from CaCO_3_.
4. Since HCO_3_^−^ is unstable in the absence of water vapor, it is released as CO.

**Figure 8:**
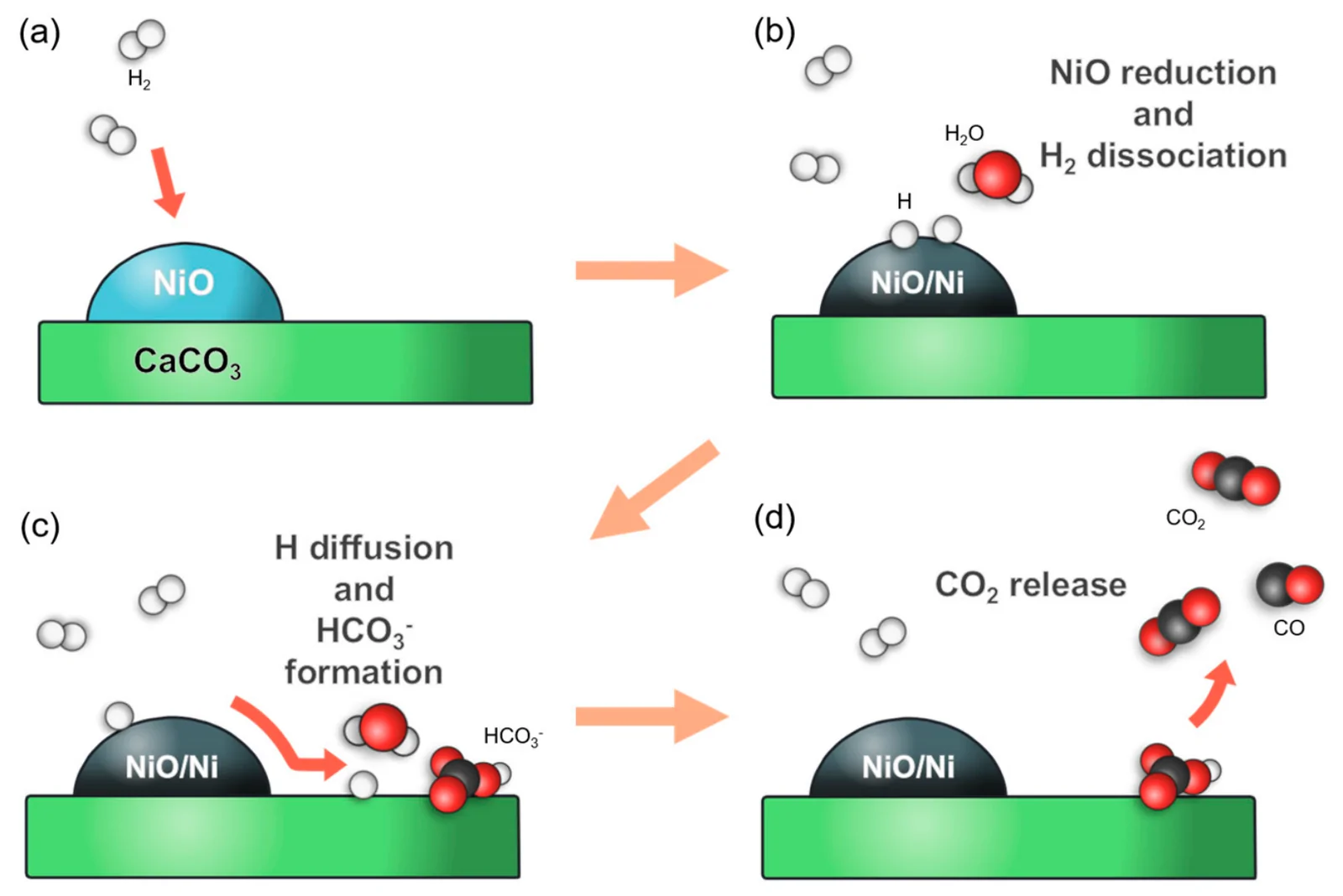
Schematic illustration of H-driven CaCO_3_ decomposition: (**a**) supply of H_2_ to NiO/CaCO_3_, (**b**) H_2_O formation via NiO reduction and H_2_ dissociation on Ni, (**c**) HCO_3_^−^ formation accompanying supply of H atoms and H_2_O from Ni to CaCO_3_, (**d**) CO_2_ and/or CO release accompanying HCO_3_^−^ decomposition. Pink arrows indicate the movement of chemical species and the progress of the reactions, while pale orange arrows indicate the temporal sequence of the reaction stages.

Under these conditions, CH_4_ generation (Equation (8)) is not active at 250 °C, and the majority of the decomposition products are detected as CO. When H_2_ is supplied sufficiently, the H_2_O generated by the NiO reduction and the CO undergo the WGS reaction on the Ni surface, producing CO_2_ and H_2_. When H_2_ supply is insufficient, the H_2_O generation is reduced due to the decreased NiO reduction rate, and consequently CO is detected without conversion as illustrated in Figure 8. In this way, H_2_ partial pressure is demonstrated to be an important parameter controlling both the overall extent of the reaction and the CO/CO_2_ selectivity. Partial pressure increase in H_2_O during the reaction, especially upon the reduction in NiO to Ni under H_2_, is crucial as well since the H_2_O can react with CaO forming Ca(OH)_2_. It is also inferred that CO_2_ generated during decomposition of CaCO_3_ can react with this Ca(OH)_2_ to regenerate CaCO_3_.

The results shown above and the foregoing discussion are consistent with the report by Giammaria and Lefferts [14], which demonstrated the catalytic effect of H_2_O on the thermal decomposition of CaCO_3_. They reported that H_2_O promotes the CaCO_3_ decomposition via formation of CaHCO_3_·OH, proceeding through Ca(OH)_2_ to CaO. By this, the apparent activation energy decreases from 201 kJ/mol to 140 kJ/mol [14]. It is therefore considered that, if formation of CaHCO_3_·OH can be achieved, thermal decomposition at low temperatures is feasible. Direct spectroscopic identification of HCO_3_^−^ was not possible: in the undiluted samples used here, the diffuse reflectance spectra are saturated around 1220 cm^−1^, where the δ(COH) band is expected, owing to the strong fundamental bands of bulk CaCO_3_, and the region around 1600 cm^−1^ overlaps with the δ(HOH) mode of adsorbed H_2_O and the ν3 mode of CO_3_^2−^; its involvement is therefore inferred from the literature [14,38,39] rather than demonstrated directly.

It is also well established that molecular hydrogen dissociates on metallic catalysts including metallic Ni and related elements to supply atomic hydrogen, which then migrates to adjacent support surfaces via hydrogen spillover [22]. Recent studies have demonstrated that this hydrogen spillover interacts with Lewis acid sites on oxide supports to generate Brønsted acid sites [38,39]. It is therefore considered that in the present system, the supply of atomic H from Ni leads to the formation of Brønsted acid species, which promote the formation of HCO_3_^−^ as an intermediate, thereby enabling CaCO_3_ decomposition at the low temperature of 250 °C. The absence of weight changes and similar events from 250 °C to the temperature range near 500 °C where CH_4_ generation is expected to occur should also be noted. This is attributed to the completion of H_2_O supply through the completion of NiO reduction.

Furthermore, according to Nikulshina et al. [40], the carbonation rate of Ca(OH)_2_ is extremely low at 200–250 °C but increases sharply above 350 °C. Therefore, even if a small degree of decomposition occurs in this temperature range, the regeneration of CaCO_3_ prevents observable weight changes in the same range. This is in line with the absence of weight change in our TG results at 200–250 °C.

Here, H_2_O supply during the reaction by oxide reduction is of key importance. Further, the resultant metallic surface and its catalytic activity for the H_2_ dissociation dominate the CH_4_ formation. Compared to Ni, Cu is known to be a poor catalyst for the H_2_ dissociation [23,41]. Comparing these two types of metal catalysts may provide insights into CH_4_ formation behavior; this will be discussed in the next section.

#### 3.3.1. CH_4_ Evolution Behavior in NiO/CaCO_3_ (TPD-MS)

**Figure 9:**
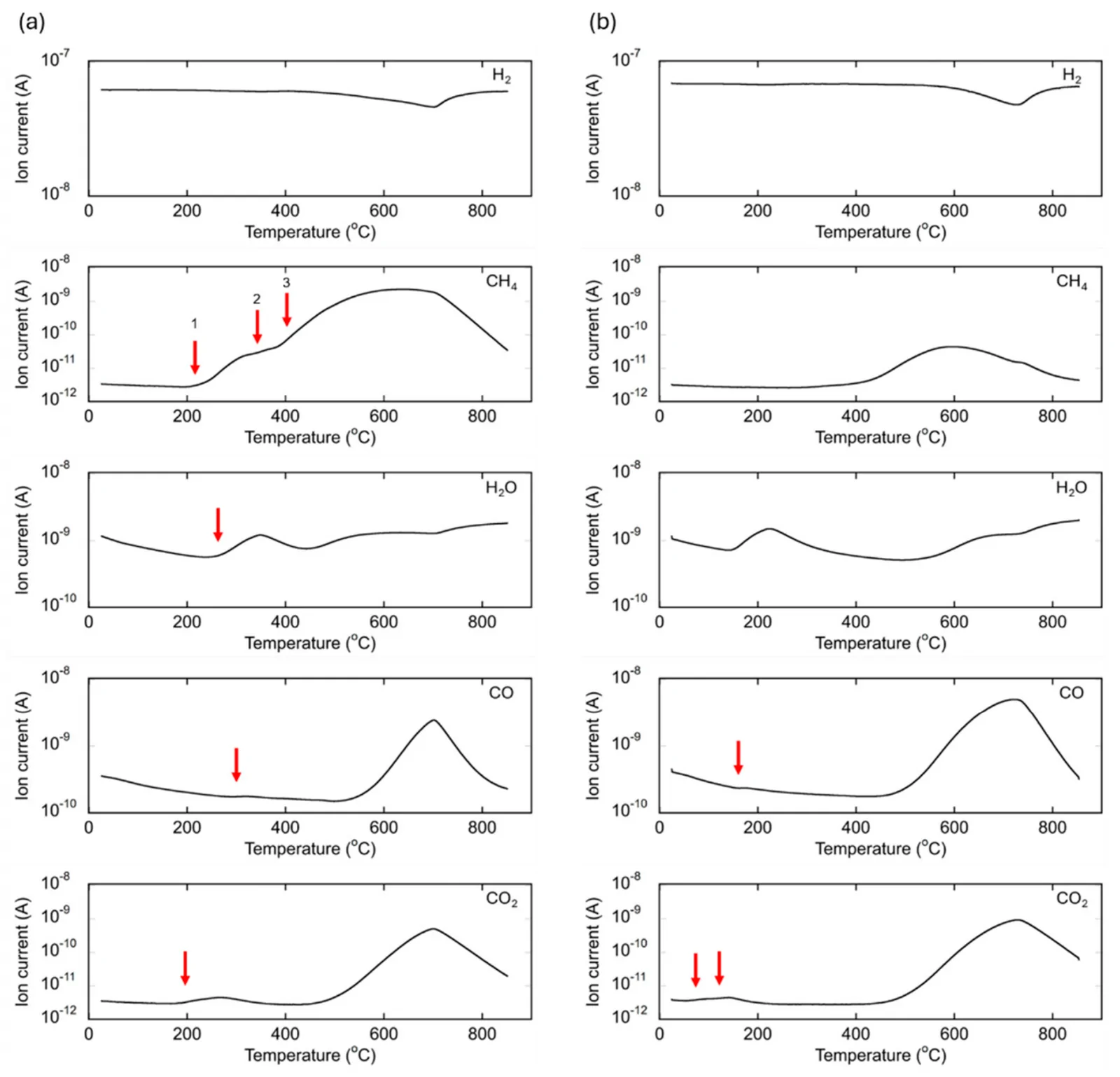
Results of TPD-MS (heating rate: 10 °C/min, H_2_ flow rate: 40 ccm). (**a**) NiO/CaCO_3_, (**b**) CuO/CaCO_3_. CH_4_ was monitored by CH_3_^+^ to differentiate from O^+^. There is a three-step increase in the CH_4_ observed; 1st at 200 °C, 2nd at 350 °C, 3rd at 400 °C (for details, see Figure S9). In (**a**), the release of CO_2_ is observed from just below 200 °C, whereas no CH_4_ formation is detected. The H_2_O signal rises at higher temperatures than that of CO_2_. This apparently delayed detection of H_2_O over CO_2_ is due to condensation of H_2_O in the reactor system. In (**b**), CO_2_ release begins at around 100 °C and exhibits a two-stage peak profile, which is attributed to a series of CuO reduction processes to Cu (see main text and Figure S9). Arrows indicate the onset points at which the signal of each species begins to increase.

Figure 9 summarizes the results of H_2_ flow TPD-MS measurements on both NiO/CaCO_3_ and CuO/CaCO_3_ (metal oxide-to-CaCO_3_ weight ratio of 1:9). The signals at *m*/*z* = 2, 15, 18, 28 and 44 were monitored for H_2_, CH_4_, H_2_O, CO and CO_2_, respectively. CH_4_ was monitored by *m*/*z* = 15 rather than at its molecular ion *m*/*z* = 16, which overlaps with the O^+^ ion. The assignment of the *m*/*z* = 15 signal to CH_4_ was confirmed by the essentially identical temperature profiles of the *m*/*z* = 14, 15 and 16 signals (Figure S3). Since CaCO_3_ is the only source of releasing CO_2_ in both of the NiO/CaCO_3_ and CuO/CaCO_3_ systems, a rise in the CO_2_ signal indicates the decomposition of CaCO_3_. This was corroborated by the TPD-MS results obtained under an Ar atmosphere; no increase in the CO_2_ signal was observed until the decomposition temperature of CaCO_3_ was reached (see Figure S10).

In the NiO/CaCO_3_ system, H_2_O signal increased at around 250 °C (Figure 9a). But, preceding this, an increase in CO_2_ signal was first observed near 200 °C. This appears to contradict the XRD result showing incomplete NiO reduction (Figure 3), as well as the earlier QMS result in which H_2_O was detected before CO_2_ at 250 °C, suggesting that CaCO_3_ decomposition apparently preceded the reduction in NiO. However, this can be explained as follows, considering H_2_O condensation in the reactor system. First, the CO_2_ release near 200 °C is followed by the evolution of CH_4_ (see the CO_2_ and CH_4_ signals in Figure 9a). Since the formation of CH_4_ requires metallic Ni, the reduction in NiO must have proceeded by this point, yielding H_2_O. But Richardson et al. demonstrated that the reduction in NiO under H_2_ proceeds from 175 °C, albeit at a slow rate, with NiO conversion reaching only approximately 20–30% even after 50 min at this temperature [42]. The reduction in our experimental system is therefore considered to have proceeded only partially, yielding a small amount of H_2_O. This H_2_O can easily be condensed in the reactor system including tubing between FBR and QMS, resulting in the apparently delayed detection of H_2_O at the QMS detector. Partial reduction in NiO at 250 °C is also consistent with the residual NiO observed by XRD (Figure 3).

The trace amount of H_2_O generated by this partial reduction in NiO is inferred to promote CO_2_ release as discussed above, with most of the H_2_O generated being trapped within the sample as Ca(OH)_2_, thereby reducing the amount of H_2_O present in the reaction gas monitored by the QMS. In addition, H_2_O could partially be condensed and adsorbed onto the walls of the tubing leading to the QMS detector so that the detection of H_2_O is delayed overall. These factors may explain the observed trend of Figure 9, where CO_2_ and CH_4_ increase first over H_2_O in the TPD-MS measurements. The H_2_O signal observed near 250 °C should therefore contain contributions both from the H_2_O accompanying CH_4_ formation via the Sabatier reaction and from the reduction in the residual NiO, and these two cannot be resolved separately.

A three-step increase in *m*/*z* = 15, the fragment ion of CH_4_ (the CH_4_ signal in Figure 9a and Figure S9a), was also confirmed. The first step, a rise below 250 °C, is attributed to the Sabatier reaction (Equation (9)) catalyzed by Ni generated by NiO reduction, proceeding in the presence of the co-generated CO_2_ [43]. The second stage, a minor signal increase near 350–400 °C, is inferred to correspond to the direct hydrogenation of CaCO_3_ driven by atomic H supplied from Ni. The third stage is the rise from approximately 400 °C, attributed to the Sabatier reaction driven by CO_2_ generated by thermal decomposition of CaCO_3_ under hydrogen atmosphere proceeding in the ordinary manner.

After the low-temperature decomposition of CaCO_3_, the CO_2_ signal attributable to CaCO_3_ decomposition increased gradually at around 500–600 °C, and the CO signal developed approximately 100 °C above this (Figure 9a). This also supports the interpretation of the third stage discussed above.

(9) $${CO}_{2}+4H_{2}↔\;{CH}_{4}+2H_{2}O\;\;\;\;\;\Delta H_{298\;K}^{o}=-165\;kJ/mol$$

The re-rise in H_2_ and H_2_O signals at around 700 °C near the end of the measurement, occurring after the CO, CO_2_, and CH_4_ signals had passed their peaks, is interpreted as follows: as the release of these species approached completion, the fraction of H_2_ consumed in reaction decreased, causing the proportion of H_2_ in the reaction gas to increase and thus more H_2_ to reach the detector, raising the signal. Additionally, water droplets were visually observed by eyes on the inner wall of the quartz tube during the experiment, suggesting that H_2_O generated along with CH_4_ generation was released with a delay, contributing to the observed H_2_O signal rise.

#### 3.3.2. CH_4_ Evolution Behavior in CuO/CaCO_3_ (TPD-MS)

Figure 9b shows the results for CuO/CaCO_3_. Given the mechanism established for the NiO/CaCO_3_ system, CO_2_ release is expected to begin at lower temperatures since the reduction in CuO starts below that of NiO and show a two-stage profile reflecting the stepwise reduction via Cu_2_O. A rise in CO_2_ was also observed in this sample from 100 °C onward. In the case of CuO, a shoulder peak suggesting a two-stage reaction is observed in this temperature range (the CO_2_ signal in Figure 9b and Figure S9b). The first stage may correspond to the release of adsorbed CO_2_ or a very small amount of CuCO_3_. However, since the thermal decomposition temperature of CuCO_3_ is 290 °C [44] and the sample was heated to 500 °C during preparation, it is expected that only trace amounts of Cu carbonate are present. Furthermore, as shown in the Ar flow TG results in Figure S1, no weight loss was confirmed at the decomposition temperature of the carbonate of the loaded element for either the NiO/CaCO_3_ or CuO/CaCO_3_ samples, leading to the conclusion that this is at most a minor CO_2_ adsorption with negligible influence here.

The first stage must, therefore, originate from the reduction process itself, and its specific origin is identified below in connection with the stepwise reduction in CuO. The second stage can be assigned more directly: it is considered to involve promotion of CaCO_3_ thermal decomposition through H_2_O generated during CuO reduction, with H diffusion from Cu playing a role—analogous to the NiO/CaCO_3_ system. As noted above, Cu is known to have a high activation barrier for H_2_ dissociation on most surface facets [23]; nevertheless, sub-nanometer Cu particles have been reported to catalyze the Sabatier reaction under elevated pressures [45], suggesting that sub-nanometer Cu particles may also exhibit limited catalytic activity under the atmospheric-pressure conditions of the present study. The observed CH_4_ generation being less pronounced than that of the NiO-loaded sample also supports this interpretation.

When comparing the hydrogen reduction in NiO and CuO (Equations (5) and (6)), the thermal balance for NiO reduction is negligible, whereas CuO reduction is markedly exothermic by 84.5 kJ/mol—unlike NiO (Equation (6)). This may have contributed significantly to the CO_2_ release, even locally. Furthermore, since CuO reduction proceeds via a two-stage pathway through Cu_2_O (Figure S9b), the observed peak shape can now be interpreted as such. That is, the first CO_2_ release peak corresponds to H_2_O generated by partial reduction to Cu_2_O together with partial further reduction to Cu, and the second CO_2_ release peak corresponds to the dominance of direct reduction to Cu with increasing temperature.

As with the NiO/CaCO_3_ system, re-rises in H_2_ and H_2_O were observed at around 700 °C. Continued analysis after reaching 850 °C showed a tendency for only H_2_O to decrease approximately 1000 s after the CO_2_ release peak from CaCO_3_ decomposition (Figure S7), leading to the conclusion that no further H_2_O-generating reactions occurred and that residual H_2_O was being detected.

Based on the results above, it is inferred that H_2_O generated by NiO reduction and atomic H diffusion from Ni cooperatively promote HCO_3_^−^ generation from CaCO_3_ and that this is further facilitated by H supply from reduced Ni, resulting in the observed release of CO and CO_2_ at 250 °C. Furthermore, the detection of CH_4_ near 300 °C reported by Padeste et al. [36] is inferred to have resulted from the Sabatier reaction-based CH_4_ generation (Equation (9)). This was driven by the CO and CO_2_ produced, with nearly all of the CO_2_ consumed, thereby precluding its detection.

While there was no change in the proportion of the peak assigned to CaCO_3_ in XPS for CuO/CaCO_3_, CO_2_ was detected from the same sample by TPD-MS. This discrepancy could likely be attributable to an increased reduction rate resulting from the sample temperature being kept at 250 °C in the case of the sample analyzed by XPS. A temperature of 250 °C is higher than the onset temperature of CuO reduction, as monitored by TPD-MS. This implies that the reduction in CuO was fast enough and completed at 250 °C, and the amount of H_2_O present and its residence time were not optimal for driving the decomposition of CaCO_3_, resulting in the XPS spectra being almost unchanged (Figure 4b). Re-carbonation of Ca species by the CO_2_ released during decomposition is also considered a contributing factor.

Giammaria et al. [14] reported that an optimal water vapor content of 1.5% promotes isothermal CaCO_3_ decomposition at 590–650 °C. In the present study, CaCO_3_ decomposition at 250 °C was confined to a portion of the sample surface. Optimizing the loading and dispersion of the metal oxide, as well as the H_2_ supply conditions, may extend this decomposition to a broader fraction of the sample.

While this literature precedent underscores the promoting role of H_2_O, the individual contributions of H_2_O and atomic H cannot be fully resolved from the present data. TPD-MS under Ar flow showed no low-temperature CO_2_ evolution for either composite (Figure S10). H_2_ is therefore necessary for the present reaction. In addition, unsupported CaCO_3_ showed no detectable change upon heating under H_2_ in the in situ DRIFTS spectra and the ex situ XRD (Figures S8c and S2). This suggests that the supported metal oxide is also necessary. The action of H_2_ thus appears to be mediated by the H_2_O generated upon oxide reduction, by atomic H supplied from the resulting metal, or by both. The uncalibrated QMS signals allow only an indicative comparison. Within this limitation, the raw *m*/*z* = 44 ion currents of the Ni system, recorded under identical conditions, were not lower than those of the Cu system by an order of magnitude or more. Moreover, carbon was released as CH_4_ only from the Ni system. This suggests, though only qualitatively, that more carbon is released from CaCO_3_ in the Ni system, consistent with the higher H_2_ dissociation ability of the supported metal. However, the Sabatier and WGS reactions (Equations (9) and (7)) provide an additional H_2_O source in the Ni system. Whether atomic H acts on CaCO_3_ directly, or indirectly via a more sustained H_2_O supply, therefore cannot be distinguished with the current experimental configuration. Separating these contributions will require a fixed-bed reactor equipped with a controlled water vapor feed.

## 4. Conclusions

In the present study, NiO/CaCO_3_ composite powder was heated under a H_2_ atmosphere in an attempt to realize low-temperature decomposition of CaCO_3_. While the thermal decomposition of CaCO_3_ ordinarily occurs above 700 °C, the present study achieved partial decomposition of CaCO_3_ in NiO/CaCO_3_ at an even lower temperature of 250 °C. This temperature range falls within the sub-300 °C regime, where unutilized industrial waste heat is most abundantly available, and the present findings provide important insights into the practical implementation of CaL.

XRD and XPS analyses confirmed the formation of Ca(OH)_2_, and probably CaO, in NiO/CaCO_3_ after heating under H_2_ atmosphere. Outlet gas analysis by QMS directly demonstrated the progress of CaCO_3_ decomposition through detection of CO and CO_2_. In contrast, the reference CuO/CaCO_3_ sample exhibited a reaction confined to a lower temperature range and occurring on a smaller scale, indicating that the present reaction is characteristic of metal oxides capable of H_2_ dissociation, with the H_2_O generated during their reduction acting as an essential co-factor. The proposed reaction mechanism involves the cooperative action of H_2_O generated by NiO reduction and atomic H supply (H spillover) from Ni, which are inferred to promote HCO_3_^−^ generation from CaCO_3_ and its release as CO. Varying the H_2_ partial pressure showed that the resulting CO is converted to CO_2_ via the WGS reaction on the Ni surface under high H_2_ concentration conditions, whereas the WGS is suppressed and CO is detected without conversion under low H_2_ concentration conditions. Although the current data do not allow quantification of this selectivity, they suggest that H_2_ supply could serve as a new control factor in process design. This indicates the potential for CaL to be operated not merely as a CO_2_ capture process, but also as a route for producing CO, a chemical feedstock. This possibility warrants further investigation.

It should be noted, however, that the CaCO_3_ decomposition confirmed in the present study was confined to the vicinity of interface regions where atomic H supply from NiO and Ni can reach. Future work should focus on optimizing NiO loading amount and dispersion, controlling H_2_ partial pressure and heating conditions, quantifying the role of H_2_O through controlled co-feeding of water vapor, establishing a quantitative carbon balance by calibration of the mass spectrometric signals against reference gases, and developing strategies to suppress recarbonation of Ca(OH)_2_ by CO_2_, with the aim of extending the low-temperature decomposition reaction to a larger fraction of the sample. Through these efforts, it is expected that a pathway toward the practical utilization of CaL driven by sub-300 °C unutilized waste heat will be established.

## Figures and Tables

**Table 1:** Powder sample preparation conditions.

	Metal Oxide:CaCO_3_ (wt. Ratio)	(CH_3_COO)_2_Ni·4H_2_O	(CH_3_COO)_2_Cu·H_2_O	CaCO_3_	Distilled Water	Analysis
**NiO/CaCO_3_**	1:9	0.424 g	-	0.900 g	50 mL	FBR
	2:8	0.848 g		0.800 g		TG
**CuO/CaCO_3_**	1:9	-	0.314 g	0.900 g		FBR
	2:8		0.628 g	0.800 g		TG
**Calcination temperature**	500 °C					

**Table 2:** Concentration of supported elements (measured by XRF vs. calculated from the weighed amounts).

	Metal Oxide:CaCO_3_ (wt. Ratio)	Experimental Value (at %)	Nominal Value (at %)
**NiO/CaCO_3_**	1:9	21.7 at%Ni	15.9 at%Ni
	2:8	41.7 at%Ni	29.9 at%Ni
**CuO/CaCO_3_**	1:9	16.8 at%Cu	14.9 at%Cu
	2:8	34.4 at%Cu	28.3 at%Cu

## Data Availability

The original contributions presented in this study are included in the article/Supplementary Materials. Further inquiries can be directed to the corresponding authors.

## Supplementary Materials

The following supporting information can be downloaded at https://www.mdpi.com/article/10.3390/ma19173795/s1. Figure S1: TG curves of the NiO/CaCO_3_ and CuO/CaCO_3_ powders under Ar flow. Figure S2: XRD patterns of unsupported CaCO_3_ powder before and after heating under H_2_ at 250 °C. Figure S3: Ni 2p_3/2_ and Cu 2p_3/2_ XPS spectra of NiO/CaCO_3_ and CuO/CaCO_3_ before and after heating at 250 °C under H_2_. Figure S4: Weight change in the NiO/CaCO_3_ composite powder (Ni:Ca = 1:1) at 250 °C under Ar–H_2_ flow after heating under Ar. Figure S5: QMS signals of the reaction gas from NiO/CaCO_3_ heated to 250 °C under Ar and then exposed to H_2_–Ar mixtures at a total flow rate of 40 ccm. Figure S6: QMS signals at *m*/*z* = 14, 15 and 16 during H_2_ flow TPD-MS of NiO/CaCO_3_. Figure S7: Time course of the QMS signals during H_2_ flow TPD-MS of CuO/CaCO_3_. Figure S8: DRIFTS spectra of NiO/CaCO_3_, CuO/CaCO_3_, and unsupported CaCO_3_ under H_2_ flow at the start of the experiment and after 60 min at 250 °C. Figure S9: Enlarged CH_4_ signal (*m*/*z* = 15) for NiO/CaCO_3_ showing the second methanation step, and CO_2_ signal (*m*/*z* = 44) for CuO/CaCO_3_ showing two-step CO_2_ evolution. Figure S10: CO_2_ signal (*m*/*z* = 44) during Ar flow TPD-MS of NiO/CaCO_3_ and CuO/CaCO_3_ (40 ccm, 10 °C/min). Table S1: Weight change ratio (wt%) upon CaCO_3_ decomposition of NiO/CaCO_3_ and CuO/CaCO_3_.
