# Supplementary material for: Low-Temperature Decomposition of CaCO3 in NiO/CaCO3 and CuO/CaCO3 Powder Under H2 at Industrial Waste Heat Temperatures
Source: Materials (Basel). 2026 Sep 6;19(17):3795. doi: 10.3390/ma19173795 (PMC13567594; doi:10.3390/ma19173795)
Supplement: Supplementary file 1 [file materials-19-03795-s001.zip › materials-4520145-supplementary.pdf]

Low-temperature decomposition of $CaCO_3$ in $NiO/CaCO_3$ and $CuO/CaCO_3$ powder under H_2 at industrial waste-heat temperatures

Tomoaki Yoshida [1,2] and Ryota Gemma [1,2]

[1] Graduate School of Science and Technology Tokai University, Japan

[2] Micro/Nano Technology Center, Tokai University, Japan

Supplementary Information

Metal oxide loading on $CaCO_3$

Figure S1 shows the TG results for the $NiO/CaCO_3$ and $CuO/CaCO_3$ powders under Ar flow. For both samples, only the weight loss corresponding to the thermal decomposition of $CaCO_3$ was observed, and the loss in each case agreed well with the calculated value. This confirms that the powders were prepared at the intended weight ratios.

In addition, no weight loss was observed at the decomposition temperatures of $NiCO_3$ (270–420 °C) or $CuCO_3$ (380 °C), confirming the absence of carbonates of the loaded elements.

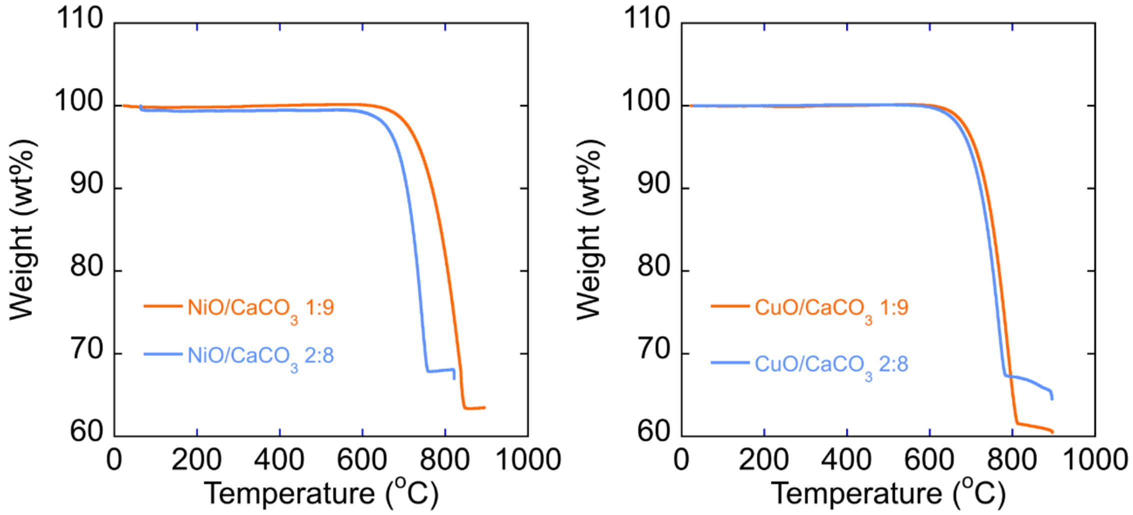

Figure S1 TG curves of the $NiO/CaCO_3$ and $CuO/CaCO_3$ powders under Ar flow.

Table S1 Weight change ratio (wt%) upon $CaCO_3$ decomposition of $NiO/CaCO_3$ and $CuO/CaCO_3$ (measured by TG vs. calculated from the weighed amounts)

	Metal Oxide:$CaCO_3$ (wt. ratio)	Experimental weight loss (wt %)	Calculated weight loss (wt%)
$NiO/CaCO_3$	1:9	37.1	38.5
	2:8	33.0	33.4
$CuO/CaCO_3$	1:9	38.4	38.6
	2:8	32.5	38.6
**Heating rate**	10 °C/min		

XRD of $CaCO_3$ heated under H_2

Figure S2 shows the XRD patterns of $CaCO_3$ powder without any metal oxide loading before and after heating under H_2 at 250 °C for 2 hours. No appreciable change was observed between the two patterns, indicating that the low-temperature decomposition of $CaCO_3$ reported here requires the loading of a metal oxide.

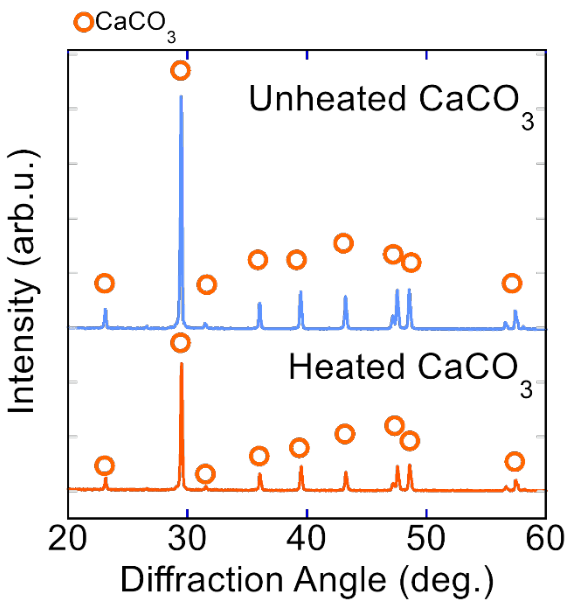

Figure S2 XRD patterns of unsupported $CaCO_3$ powder before and after heating under H_2 at 250 °C for 2 h. No appreciable change was observed, indicating that the low-temperature decomposition requires the loading of metal oxide.

Reduction of NiO and CuO confirmed by XPS

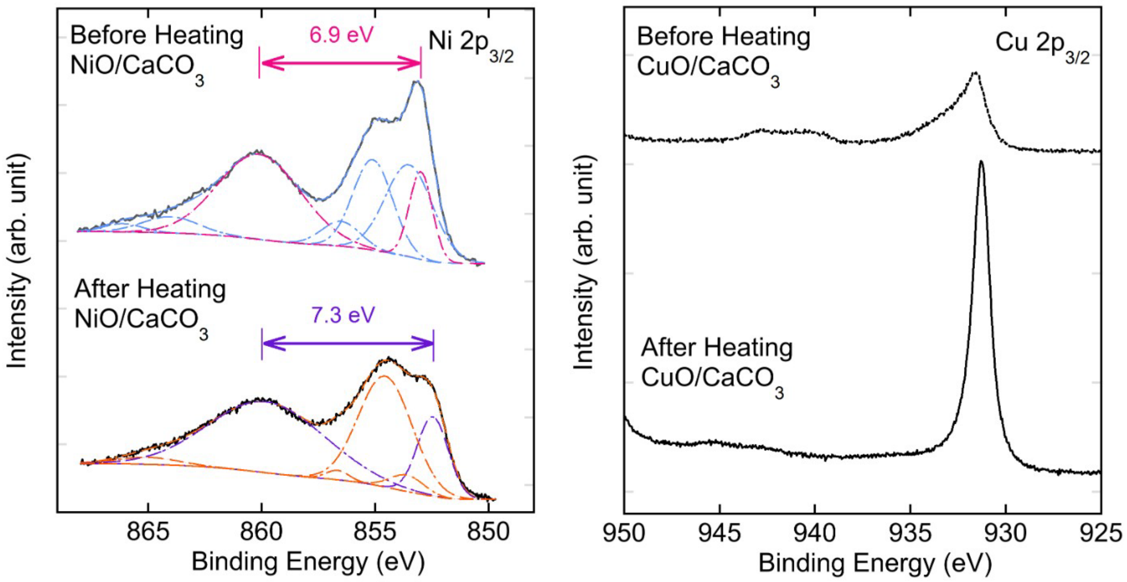

Figure S3 Ni $2p_{3/2}$ and Cu $2p_{3/2}$ XPS spectra of $NiO/CaCO_3$ and $CuO/CaCO_3$ before and after heating at 250 °C under H_2.

Figure S3 shows the Ni $2p_{3/2}$ and Cu $2p_{3/2}$ spectra of $NiO/CaCO_3$ and $CuO/CaCO_3$ before and after heating at 250 °C for 2 h under H_2 atmosphere. For $NiO/CaCO_3$, the spectrum before heating is composed of the multiplet-split main peak and the shake-up satellite characteristic of NiO, with a separation of 6.9 eV between the main peak maximum and the satellite. After heating, a new component appears at ca. 852.5 eV, close to the value reported for metallic Ni, which increases the apparent main-to-satellite separation to 7.3 eV. The coexistence of this metallic component with the remaining NiO-derived features indicates that the reduction of NiO proceeded partially under these conditions, consistent with the residual NiO observed by XRD (Figure 3). For $CuO/CaCO_3$, the shake-up satellite at 940–945 eV, which is characteristic of Cu^{2+}, disappears almost completely after heating, indicating the reduction of CuO. Since Cu^0 and Cu^+ cannot be distinguished from the Cu $2p_{3/2}$ binding energy alone, the formation of metallic Cu is confirmed by XRD (Figure 3).

Weight loss by NiO reduction estimated by TG

Figure S4 shows the weight change of the NiO/$CaCO_3$ composite powder (Ni:Ca molar ratio = 1:1) heated to 250 °C under Ar and subsequently switching to Ar–H_2 flow (Ar 225 ccm, H2 25 ccm). Complete reduction of the NiO by H_2 in this sample corresponds to a weight loss of 9.15 wt%. A larger loss would indicate concurrent decomposition of $CaCO_3$; however, the measured value was 9.27 wt%, and the slight excess over 9.15 wt% falls within the range of measurement uncertainty.

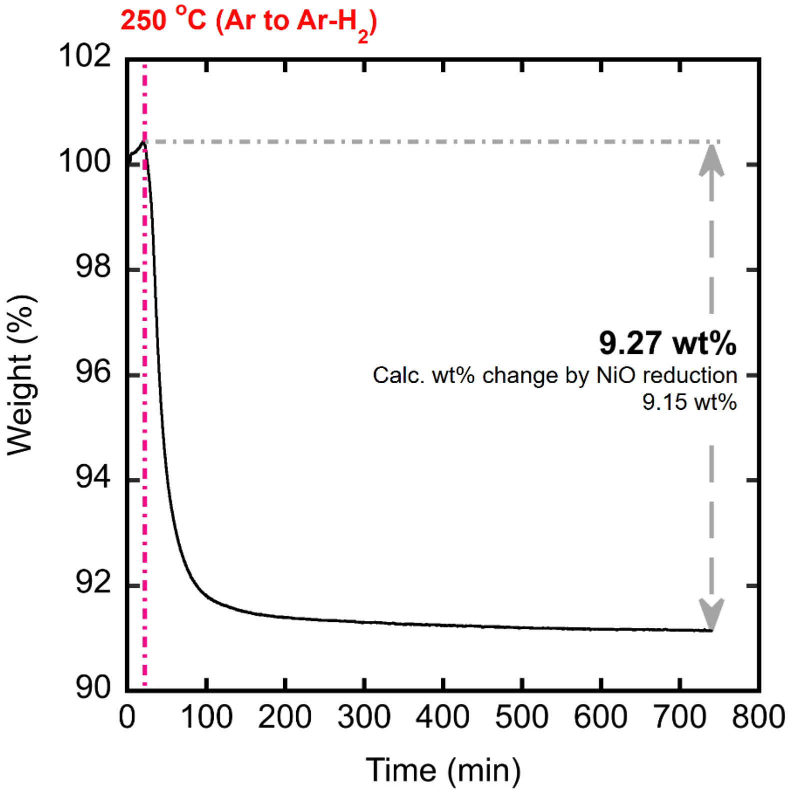

Figure S4 Weight change of the NiO/$CaCO_3$ composite powder (Ni:Ca = 1:1) at 250 °C under Ar–H_2 flow after heating under Ar. The loss corresponds to complete NiO reduction (9.15 wt%) within error.

This excess can be used to estimate the extent of $CaCO_3$ conversion, subject to the assumptions stated below. For reference, complete decarbonation of the $CaCO_3$ in this composite ($CaCO_3 \rightarrow CaO + CO_2$) would correspond to a weight loss of 25.2 wt% on the same basis. Assuming that the reduction of NiO was complete and that the entire excess of 0.12 wt% originates solely from the transformation of $CaCO_3$, the excess corresponds to a conversion of ca. 0.8 % if $Ca(OH)_2$ is

formed ($CaCO_3 + H_2 \rightarrow Ca(OH)_2 + CO_2$) or ca. 0.5 % if CaO is formed. Two caveats apply to this estimate. On one hand, the excess may include contributions from the desorption of H_2O produced during NiO reduction and from physisorbed water, in which case the actual conversion would be smaller. On the other hand, as shown in Figure 3, the reduction of NiO does not proceed to completion under these conditions; if part of the NiO remained unreduced, the weight loss assigned to the reduction would be overestimated and the conversion of $CaCO_3$ correspondingly underestimated, so that the actual value could exceed 1 %. The present data do not allow these contributions to be resolved, and the values above should be regarded as an order-of-magnitude estimate under the stated assumptions rather than a strict bound.

Mass spectrometry of the reaction gas at 250ºC

Figure S5 shows the QMS analysis results for the reaction gas obtained when the NiO/$CaCO_3$ composite powder was heated to 250ºC under Ar flow and part of the flowing gas was then

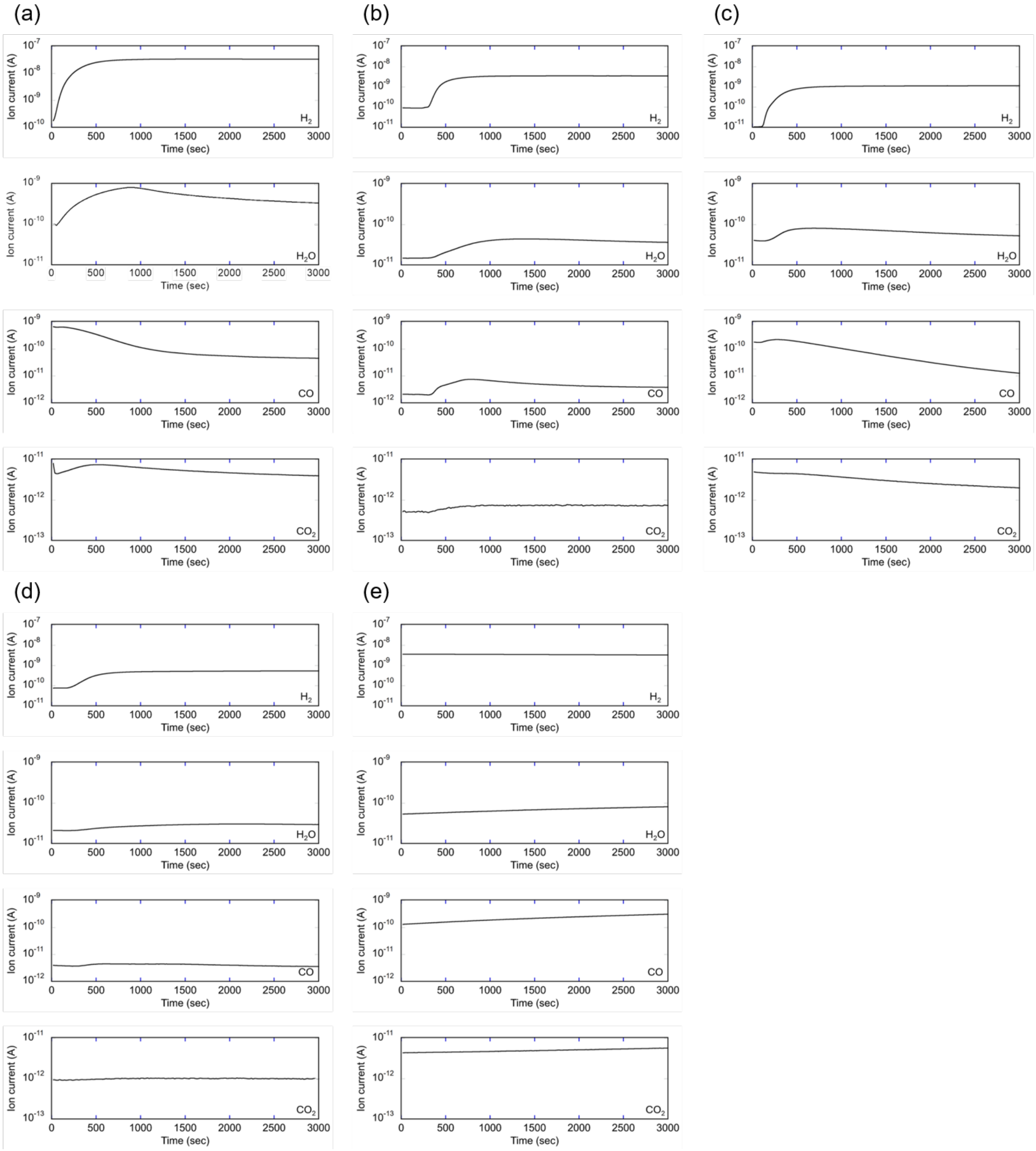

Figure S5 QMS signals of the reaction gas from NiO/$CaCO_3$ heated to 250 ºC under Ar and then exposed to H_2–Ar mixtures at a total flow rate of 40 ccm: (a) H_2 40 ccm, (b) H_2 30 ccm/Ar 10 ccm, (c) H_2 20 ccm/Ar 20 ccm, (d) H_2 10 ccm/Ar 30 ccm, and (e) Ar 40 ccm. CO or CO_2 did not show characteristic increase under Ar alone (e).

switched to H_2. As the H_2 concentration increased, the CO/CO_2 signal ratio in the reaction gas shifted toward CO_2, whereas the CO signal became relatively prominent as the H_2 concentration decreased. Under Ar flow alone (Figure S5e), neither CO nor CO_2 was detected, confirming that the reaction is driven by H_2.

Assignment of the m/z = 15 signal to CH4 confirmed by TPD-MS

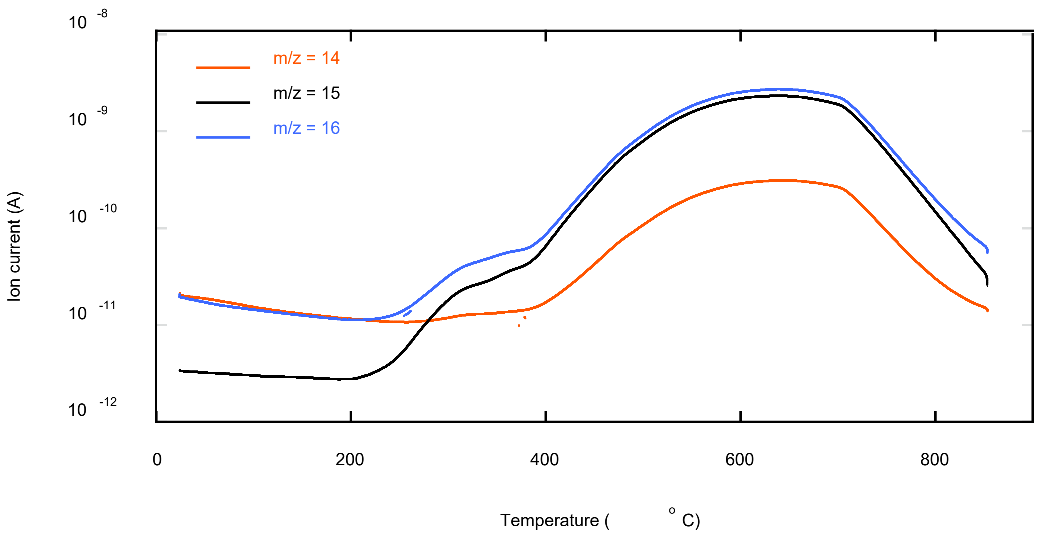

Figure S6 m/z = 14 (CH_2^+), 15 (CH_3^+) and 16 (CH_4^+) signals recorded for NiO/$CaCO_3$ during the H_2 flow TPD-MS measurement shown in Figure 9. The m/z = 15 and 16 signals show closely similar profiles above ca. 250 °C, while the m/z = 14 signal remains at the background level of ca. 10^{-11} A below 400 °C and follows the other two signals over the main evolution range above 400 °C.

Figure S6 shows the m/z = 14, 15 and 16 signals recorded for NiO/$CaCO_3$ during the H_2 flow TPD-MS measurement shown in Figure 9. Above ca. 250 °C, the m/z = 15 and 16 signals exhibit closely similar temperature profiles over the entire evolution range, including the intermediate step at 300–380 °C and the main peak centered around 650 °C, indicating that the two signals share a common origin, i.e., the CH_3^+ and CH_4^+ fragments of CH_4. The slight excess of the m/z = 16 signal, most visible in the 250–400 °C region, is attributed to O^+ fragments of the H_2O, CO and CO_2 evolving in the same range. The m/z = 14 signal (CH_2^+, expected at ca. 16% of the m/z = 16 intensity) remains at its background level of ca. 10-11 A below 400 °C, where the expected CH_4-derived contribution falls below this background, and follows the m/z = 15 and 16 signals over the main evolution range above 400 °C. The consistent behavior of the

three fragments supports the assignment of the evolved species to CH_4. The m/z = 15 signal, which is free from interfering fragments, was therefore used to monitor CH_4 throughout this work.

Delay of the H_2O signal rise in TPD-MS

Figure S7 shows the TPD-MS results for the CuO/$CaCO_3$ sample presented in the main text, plotted against time. The behavior of the H_2O signal is of particular note. The temperature reached 700°C at approximately 5000 s. As the thermal decomposition of $CaCO_3$ ended, the CO_2 signal decreased, and the H_2 and H_2O signals increased owing to the change in partial pressure. The H_2O signal began to decrease after reaching at 850 °C (after 5500 s). At this point, water droplets were observed on the inner wall of the quartz tube.

These results suggest that the rise in the H_2O signal after the CO_2 peak maximum in Figure 10 (main text) arises from residual H_2O droplets that reached the detector as water vapor after the reaction, raising the relative partial pressure of water vapor. This also indicates that a certain time lag can occur between the generation of water vapor and the rise in the H_2O signal.

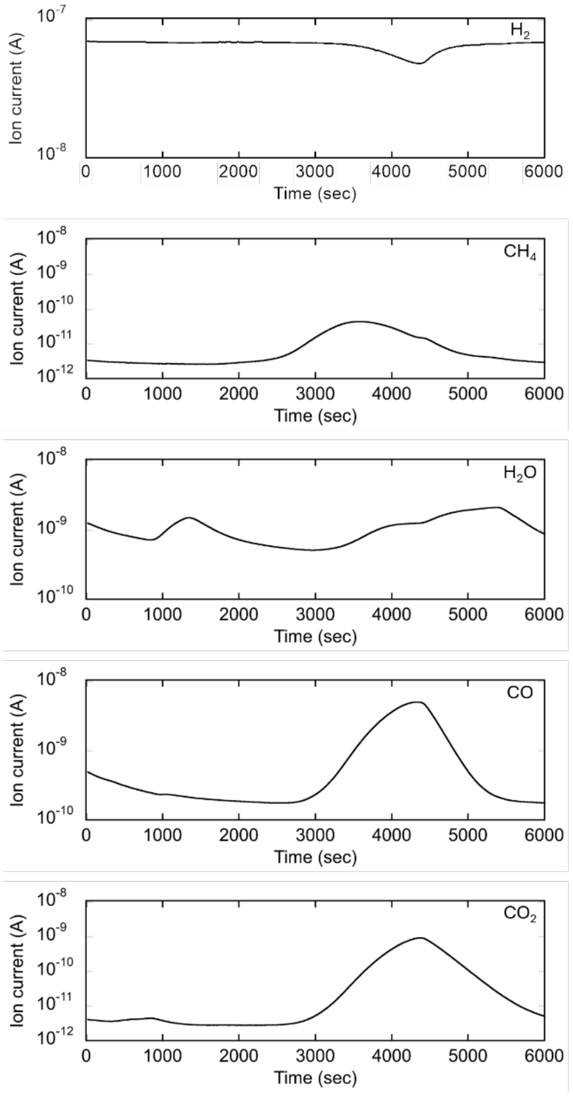

Figure S7 Time course of the QMS signals during H_2 flow TPD-MS of CuO/$CaCO_3$. The temperature reached 700 ^oC at approximately 5000 s, and the H_2O signal began to decrease some time after reaching 850 ^oC (after 5500 s).

Reflectance of metallic Ni and Cu

The reflectance R is given by the following equation.

$$R = \frac{(n-1)^2 + k^2}{(n+1)^2 + k^2}$$

At 1774 cm^{-1}, the optical constants are n = 4.11, k = 20.2 for Ni and n = 4.44, k = 30.0 for Cu, respectively. Substituting these values into the equation above gives reflectance R at 1774 cm^{-1} of 0.9621 for Ni and 0.9808 for Cu, respectively. Using these normal-incidence reflectance values, an approximate calculation was performed with the following Kubelka–Munk function:

$$f(R) = \frac{(1-R)^2}{2R}$$

$$f(R_{Cu}) = \frac{(1-0.9808)^2}{2 \times 0.9808} \approx 1.88 \times 10^4$$

$$f(R_{Ni}) = \frac{(1-0.9621)^2}{2 \times 0.9621} \approx 7.47 \times 10^4$$

$$f(R_{Ni}) - f(R_{Cu}) = 5.59 \times 10^4$$

The difference in the Kubelka–Munk function values of Ni and Cu at 1774 cm^{-1} is thus only 5.59×10^{-4}. This is more than three orders of magnitude smaller than the observed peak-intensity difference of 0.15, showing that optical artifacts of the metals are negligible. The DRIFTS results in Figure 5(b) therefore reflect a clear difference of absorption by CO_3^{2-}, where enhanced decomposition of $CaCO_3$ is suggested in NiO-loaded sample by H_2 dissociation on Ni surfaces created by NiO reduction.

Change in IR spectra before and after the experiment

Figure S8 shows the DRIFTS results for NiO/$CaCO_3$, CuO/$CaCO_3$, and $CaCO_3$ at the start of the experiment and after 60 min at 250 °C. The results for NiO/$CaCO_3$ and CuO/$CaCO_3$ are those shown in Figure 5(b). For $CaCO_3$ alone under H_2 flow, no decrease in CO_3^{2-} associated with low-temperature decomposition was observed upon heating under H_2.

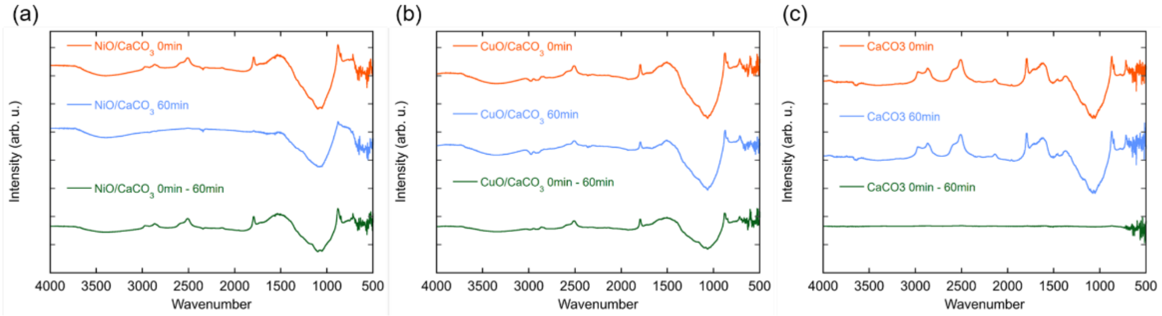

Figure S8 DRIFTS spectra of (a) NiO/$CaCO_3$, (b) CuO/$CaCO_3$, and (c) unsupported $CaCO_3$ under H_2 flow at the start of the experiment and after 60 min at 250 °C.

Note that no decrease in the CO_3^{2-} band (1791 cm^{-1}) was observed for unsupported $CaCO_3$.

Enlarged TPD-MS Profiles of CH_4 and CO_2 Signals

Figure S9 (b) shows an enlarged view of the CO_2 signal (m/z = 44) of Figure 9 (b) over the range of 50-250 °C. The increase in the CO_2 signal is found to consist of two peaks. The former peak is presumably associated with the low-temperature decomposition of $CaCO_3$ that accompanies the conversion of CuO to Cu_2O, whereas the latter peak is attributed to the overlap of the low-temperature decomposition of $CaCO_3$ accompanying the reduction of CuO to Cu and that accompanying the reduction of the Cu_2O formed in the preceding stage to Cu.

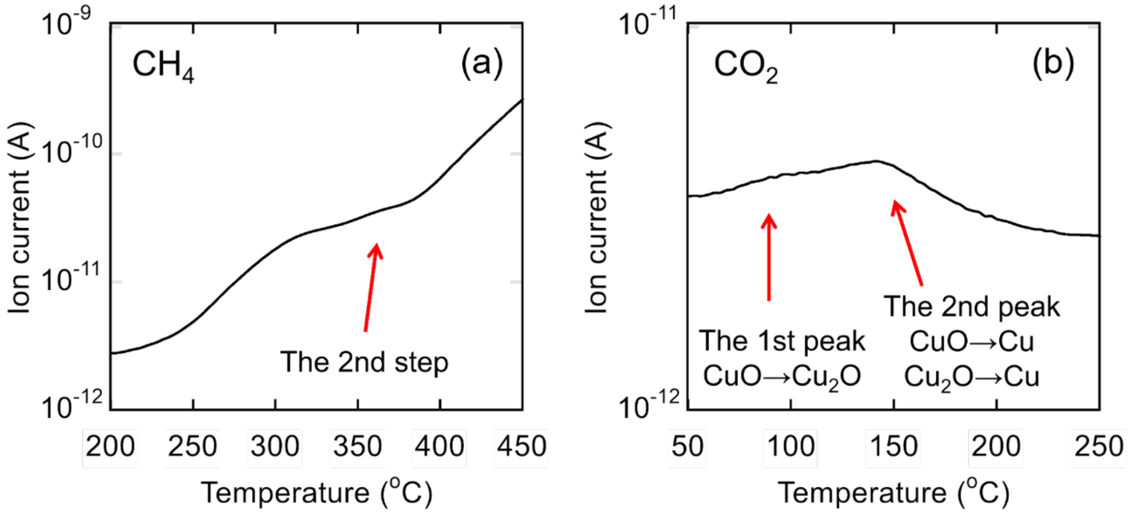

Figure S9 (a) shows an enlarged view of the CH_4 signal (m/z = 15) in Figure 9 (a) over the range of 200-450 °C. A small but distinct increase in the signal is observed at the position labeled "The 2nd step" in the figure. As described in the main text, this increase is attributed to the direct methanation of $CaCO_3$.

CO_2 signal (m/z = 44) as a function of temperature during TPD-MS under Ar flow

Figure S10 shows the CO_2 signal (m/z = 44) recorded as a function of temperature during TPD-MS under Ar flow (40 ccm, 10 °C/min), which was performed as a control for the H_2-flow TPD-

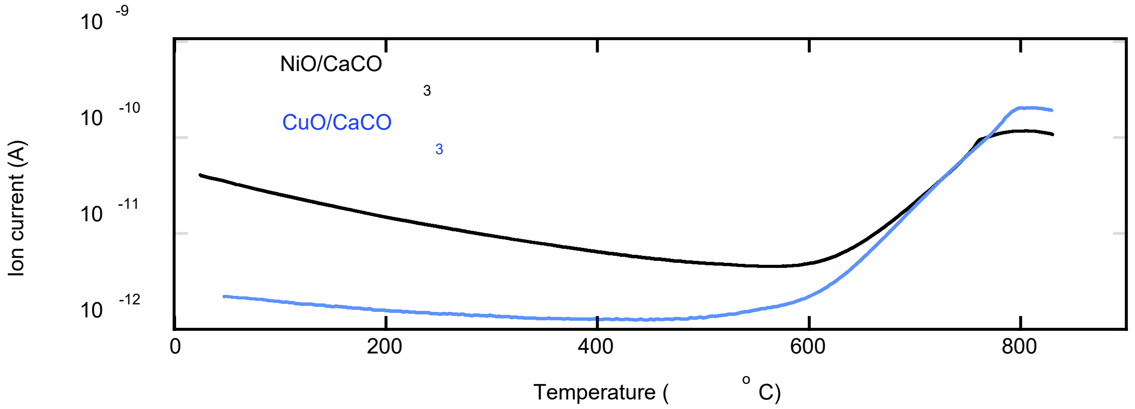

Figure S10 CO_2 signal (m/z = 44) during Ar-flow TPD-MS of NiO/$CaCO_3$ and CuO/$CaCO_3$ (40 ccm, 10 °C/min). No low-temperature CO_2 evolution was observed, in contrast to the H_2-flow results (see Fig. 10 in the main text).

MS measurements of NiO/$CaCO_3$ and CuO/$CaCO_3$ shown in Figure 9. Except for the increase near 600 ^oC, which is ascribed to the onset of $CaCO_3$ decomposition, no features other than baseline drift were detected in the low-temperature region, in contrast to the CO_2 signals observed in Figure 9.

These results indicate that the low-temperature decomposition of $CaCO_3$ reported here corresponds to the genuine decomposition of a fraction of the $CaCO_3$, rather than to an apparent decomposition arising from the formation of species such as $CuCO_3$ or $NiCO_3$ by the Cu and Ni contained in the prepared samples, or from the release of adsorbed CO_2.
